# Identification of *in vitro* and *in vivo* disconnects using transcriptomic data

**DOI:** 10.1186/s12864-015-1726-7

**Published:** 2015-08-18

**Authors:** Martin Otava, Ziv Shkedy, Willem Talloen, Geert R Verheyen, Adetayo Kasim

**Affiliations:** Interuniversity Institute for Biostatistics and Statistical Bioinformatics, Hasselt University, Martelarenlaan 32, Hasselt, 3500 Belgium; Janssen, Pharmaceutical companies of Johnson & Johnson, Turnhoutseweg 30, Beerse, 2340 Belgium; Thomas More Kempen, Geel, 2440 Belgium; Wolfson Research Institute for Health and Wellbeing, Durham University, University Boulevard, TS17 6BH Thornaby, Stockton-on-Tees, UK

**Keywords:** *in vitro*, *in vivo*, Toxicogenomics, Gene expression, Dose-response relationship, Liver toxicity

## Abstract

**Background:**

Integrating transcriptomic experiments within drug development is increasingly advocated for the early detection of toxicity. This is partly to reduce costs related to drug failures in the late, and expensive phases of clinical trials. Such an approach has proven useful both in the study of toxicology and carcinogenicity. However, general lack of translation of *in vitro* findings to *in vivo* systems remains one of the bottle necks in drug development. This paper proposes a method for identifying disconnected genes between *in vitro* and *in vivo* toxicogenomic rat experiments. The analytical framework is based on the joint modeling of dose-dependent *in vitro* and *in vivo* data using a fractional polynomial framework and biclustering algorithm.

**Results:**

Most disconnected genes identified belonged to known pathways, such as drug metabolism and oxidative stress due to reactive metabolites, bilirubin increase, glutathion depletion and phospholipidosis. We also identified compounds that were likely to induce disconnect in gene expression between *in vitro* and *in vivo* toxicogenomic rat experiments. These compounds include: sulindac and diclofenac (both linked to liver damage), naphtyl isothiocyanate (linked to hepatoxocity), indomethacin and naproxen (linked to gastrointestinal problem and damage of intestines).

**Conclusion:**

The results confirmed that there are important discrepancies between *in vitro* and *in vivo* toxicogenomic experiments. However, the contribution of this paper is to provide a tool to identify genes that are disconnected between the two systems. Pathway analysis of disconnected genes may improve our understanding of uncertainties in the mechanism of actions of drug candidates in humans, especially concerning the early detection of toxicity.

**Electronic supplementary material:**

The online version of this article (doi:10.1186/s12864-015-1726-7) contains supplementary material, which is available to authorized users.

## Background

### Introduction

Pharmaceutical companies are facing an urgent need to increase their lead compound and clinical candidate portfolios, to satisfy market demands for continued innovation and revenue growth [1]. A relatively small number of drugs are being approved, while research costs are increasing, patents are expiring, and both governments and health insurance companies are pushing for cheaper medications [2]. Moreover, 20–40 % of novel drug candidates fail because of safety issues [3, 4], increasing the costs of bringing new drugs to the market [5]. Drug development costs could be reduced substantially if undesirable toxicity of a drug candidate could be predicted at earlier stages of the drug development process [6]. Integrating transcriptomics within drug development pipelines is being increasingly considered to help the early discovery of potential safety issues during preclinical phase and toxicology studies [7–10]. Such an approach has proven useful both in toxicology [11, 12] and carcinogenicity studies [13, 14].

Toxicogenomics studies mostly focus on network building for rat *in vivo* experiments [15] or the connection between rat *in vivo* and human *in vitro* transcriptomics experiments, particularly in relation to drug induced liver injury (e.g., [16–18]). Zhang et al. [19] developed a consensus early response toxicity signatures of *in vitro* and *in vivo* toxicity in human and rat using time-dependent gene expressions. For the hepatotoxicant hydrazine, Timbrell et al. [20] reported that the effects on various parameters do not always show a quantitative or qualitative correlation between *in vivo* and *in vitro* gene signatures. Enayetallah et al. [4] profiled nine compounds for *in vitro* and *in vivo* cardiotoxicity, and reported that while there were common biological pathways for *in vivo* and *in vitro* rat experiments for drugs like dexamethasone, most of the biological pathways identified *in vivo* for the drug amiodarone were not detected *in vitro*. Early prediction of safety issues for hit or lead compounds would benefit not only from consensus signatures, but also from *disconnect* signatures between *in vivo* and *in vitro* toxicogenomics experiments. These disconnect signatures can indicate which biological pathways are less likely to translate from a simplified *in vitro* model to a complex and holistic *in vivo* system.

Toxicity signatures developed from *in vitro* models most probably reflect protein modulations or pathway changes resulting from direct effects of compounds upon cells instead of the more complex interactions found in *in vivo* systems. *In vitro* signatures could also show excessive toxicity not to be detected *in vivo* due to compensatory mechanisms found in *in vivo* systems. Thus the framework is proposed to detect genes that are disconnected between *in vitro* and *in vivo* dose-dependent toxicogenomics experiments using fractional polynomial models. Biclustering is applied to find subsets of disconnected genes that are common to several compounds. Finally, the identified groups of disconnected genes are interpreted by their most probable biological pathways.

### Data set

**Fig. 1:**
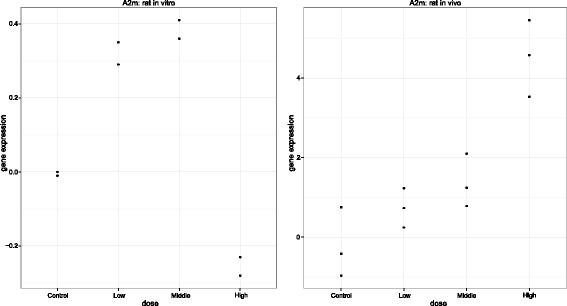
Data set example: Using gene *A2m* and compound sulindac. Observed gene expression profile for gene *A2m* under the activity of sulindac. Left panel: *in vitro* data. Right panel: *in vivo* data

The ’Toxicogenomics Project - Genomics Assisted Toxicity Evaluation system’ (TG-GATEs, TGP, [21]) is a collaborative initiative between Japanese governmental bodies and fifteen pharmaceutical companies. It offers a rich source of transcriptomics data related to toxicology, providing human *in vitro* experiments together with *in vitro* and *in vivo* rat experiments [22–24]. We focus on a subset of the TG-GATEs data set consisting of 128 therapeutic drugs from a wide range of chemotypes. Gene expression were quantified using Affymetrix chip Rat 230_2 arrays. Six weeks old male Sprague-Dawley rats were used for both experiments and a single dose study design was used. Each rat was administered a placebo (the vehicle) or one of three active doses of a compound. For *in vivo* experiment, the rats were sacrificed after a fixed time period and liver tissue was subsequently profiled for gene expression. For the *in vitro* experiments, a modified two-step collagenase perfusion method was used to isolate liver cells from 6-week-old rats. These primary cultured hepatocytes were then exposed (in duplo) to a compound and gene expression changes were investigated at multiple time points. The analysis in this manuscript focuses on gene expression data at single time point, after exposure to a therapeutic drug for 24 hours, as gene expression signals are likely to be stronger at this time point in a single-dose study design [18]. The final data set for the rat *in vitro* experiments contains 5,914 genes and 1024 arrays (2 arrays per dose per compound), while the data set for the *in vivo* experiments contains 5,914 genes and 1536 arrays (3 arrays per dose per compound). The gene expression data were pre-filtered using *I/NI calls* to minimise false positives [25, 26]. The actual response variable represents the fold change of log2 mRNA intensities between the doses and the control dose. Hereafter, referred to as ’gene expression’ for simplicity. An example of a dose-response profile of gene *A2m* for compound sulindac is shown in Fig. 1.

## Methods

A flexible fractional polynomial modeling framework is proposed to: (1) identify genes with significant dose-response relationships in an *in vitro* or *in vivo* experiments and (2) identify genes that are disconnected between the two systems. The *in vitro* and *in vivo* gene expression matrices were analysed jointly by compound and the resulting disconnected genes from the separate analyses were integrated using the *Bimax* biclustering algorithm [27] in order to identify subsets of disconnected genes that are common to several compounds.

### The fractional polynomial framework

The fractional polynomial modeling framework aims to capture non-linear relationship between a predictor and a response variable. It assumes that most non-linear profiles can be captured by a combination of two polynomial powers [28]. It is particularly appealing for modeling dose-response relationships since it does not impose monotonicity apparent in most dose-response modeling methods [29, 30]. For a single gene, let *Y*_*ij*_ denote the gene expression from an *in vitro* experiment, where *i*=1,2,…,*m* represents dose levels and *j*=1,2,…,*n*_*i*_ denotes the number of replicates per dose. The fractional polynomial framework assumes that relationships between gene expression and the compound dose can be captured by a polynomial function;

(1) $$  Y_{ij}= \beta_{0} + \beta_{1} \cdot f_{ij}(p_{1})+ \beta_{2} \cdot g_{ij}(p_{1}, p_{2}) + \varepsilon_{ij},  $$

where *ε*_*ij*_∼*N*(0,*σ*^2^) and the polynomial powers *p*_1_,*p*_2_∈*P*, where *P*={−3,−2.5,…,1.5,2}. This range of values provides enough flexibility to capture different forms of dose-response profile [28]. The functions *f*_*ij*_(*p*_1_) and *g*_*ij*_(*p*_1_,*p*_2_) are defined as

$$ f_{ij}(p_{1}) = \left\{ \begin{array}{ll} i^{p_{1}} &\; \; \; p_{1} \neq 0, \\ \log(i) &\; \; \; p_{1} = 0, \end{array} \right. $$

and

(2) $$ g_{ij}(p_{1}, p_{2}) = \left\{ \begin{array}{ll} i^{p_{2}}& p_{2} \neq p_{1},\; p_{2} \neq 0, \\ \log(i)\cdot i^{p_{2}} & p_{1} = p_{2},\; p_{2} \neq 0, \\ \log(i) & p_{2} \neq p_{1},\; p_{2} = 0, \\ \log(i)\cdot \log(i) & p_{2} = p_{1} = 0. \\ \end{array} \right.  $$

**Fig. 2:**
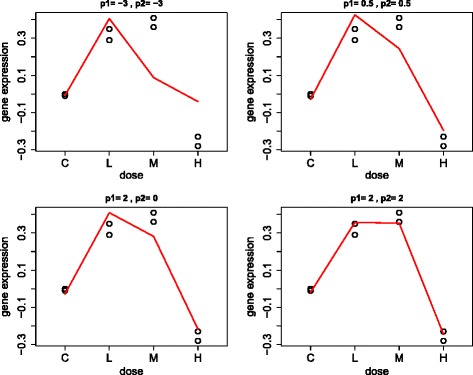
Fractional polynomial framework example: Using gene *A2m* and compound sulindac. Illustration of changes in predicted profiles by fitting fractional polynomial models with different powers on the same gene expression data. The model in the bottom right panel with *p*_1_,*p*_2_=2 is the best predictive model with the minimum Akaike’s Information Criterion (AIC)

Note that for *p*_1_≠0, *p*_2_≠0 and *p*_1_≠*p*_2_, the fractional polynomial model is given by $Y_{\textit {ij}}= \beta _{0} + \beta _{1} \cdot i^{p_{1}}+ \beta _{2} \cdot i^{p_{2}} + \varepsilon _{\textit {ij}}\phantom {\dot {i}\!}$. An example of fitting different combinations of powers for one particular gene is shown in Fig. 2.

Akaike’s information criterion (AIC, [31]) is used to select the optimal combination of *p*_1_ and *p*_2_ that best reflects the observed dose-response relationship. Optimal solutions are denoted by $\{\hat {\phi }_{1},\hat {\phi }_{2}\} = \left \{ \{p_{1}, p_{2}\} \in P, \text {AIC}(\hat {\phi }_{1},\hat {\phi }_{2}) = \min [\text {AIC}(p_{1},p_{2})] \right \}$. In order to identify genes with a significant dose-response relationship *in vitro*, a likelihood ratio test (LRT, [32]) is used to compare model (1), that best fits the data and model (3), the null model that assumes no dose effect:

(3) $$  Y_{ij}= \beta_{0} + \varepsilon_{ij}.  $$

This additional testing is necessary to adjust for the relativity of the minimum AIC criterion.

To identify disconnected genes when comparing *in vitro* and *in vivo* data, the optimal fractional polynomial function selected per gene (with $\hat {\phi }_{1},\hat {\phi }_{2}$, as fixed above) from *in vitro* data set is projected to *in vivo* data set under the assumptions that both *in vitro* and *in vivo* dose-response relationships are similar. For a single gene, let *X*_*ijk*_ denote gene expression *in vitro* and *in vivo*, where *i*=1,2,…,*m* represents dose levels, *j*=1,2,…,*n*_*i*_ denotes number of replicates per dose and *k*=1 or *k*=2 depending on whether the data is from *in vitro* or *in vivo* experiment. The *in vitro* - *in vivo* projected fractional polynomial model is specified as

(4) $$  X_{ijk} = \beta_{0} + \beta_{1} \cdot f_{ijk}(\hat{\phi}_{1}) + \beta_{2} \cdot g_{ijk}(\hat{\phi}_{1},\hat{\phi}_{2}) + \varepsilon_{ijk},  $$

where *ε*_*ijk*_∼*N*(0,*σ*^2^). A LRT is used to quantify the dissimilarity in *in vivo* - *in vitro* dose-response relationships. It compares model (4), which assumes that dose-response relationships from *in vitro* and *in vivo* experiments are the same, with model (5), which assumes different dose-response relationships.

(5) $$ X_{ijk} = \left\{ \begin{array}{lllllll} \beta_{0} & +& \beta_{1} \cdot f_{ijk}(\hat{\phi}_{1}) &+& \beta_{2} \cdot g_{ijk}(\hat{\phi}_{1},\hat{\phi}_{2}) &+& \varepsilon_{ijk} \quad \textit{in vitro},\\ (\beta_{0} + \gamma_{0}) &+& (\beta_{1} + \gamma_{1}) \cdot f_{ijk}(\hat{\phi}_{1}) &+& (\beta_{2} + \gamma_{2}) \cdot g_{ijk}(\hat{\phi}_{1},\hat{\phi}_{2}) &+& \varepsilon_{ijk} \quad \textit{in vivo}. \end{array} \right.  $$

**Fig. 3:**
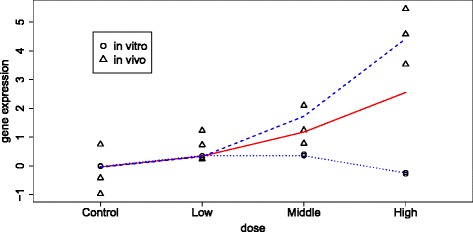
Projected fractional polynomial framework example: Using gene *A2m* and compound sulindac. Illustration of a projected fractional polynomial model from one system to the other. Red solid line shows the projected fractional polynomial model. The blue lines shows the results of fitting fractional polynomial models with different parameters for *in vitro* (dotted line) and *in vivo* data (dashed line), respectively

The comparison translates into testing if *γ*_0_=*γ*_1_=*γ*_2_=0 in model (5). An example of a projected fractional polynomial model is shown in Fig. 3. A significant result obtained from LRT comparison of model (4) and model (5) can be interpreted as a disconnect in gene expression between *in vitro* and *in vivo* rat experiments. The significance level was specified as 10 *%* after correction for multiplicity [33]. Resulting disconnected genes were subjected to fold change filtering by excluding genes with maximal dose-specific fold change between *in vitro* and *in vivo* data set less than 1. The fold change filtering further reduces false positives due to small variance genes [34, 35].

### Biclustering of genes and compounds

A biclustering framework was introduced in order to find subsets of genes and conditions with a similar pattern [36]. Biclustering methods [37, 38] are designed to cluster in two dimensions simultaneously to produce sub-matrices of the original data that behave consistently in both dimensions. The resulting sub-matrices are called biclusters. Based on the identified disconnected genes from the fractional polynomial models, a disconnect matrix *D*_(*G*×*C*)_ of binary values was created with *gc*th such that:

(6) $$  D_{gc} = \left\{ \begin{array}{ll} 1 & \text{if gene \textit{g} is disconnected for compound}\,\, c, \\ 0 & \text{otherwise}, \end{array} \right.  $$

where *G* is the number of genes that are significant for at least one compound (i.e., *G*≤5914) and *C*=128 is the number of compounds. The Bimax algorithm [27] for binary data is applied to the disconnect matrix (*G*) to find subsets of the disconnected genes that are common to several compounds.

## Results

**Fig. 4:**
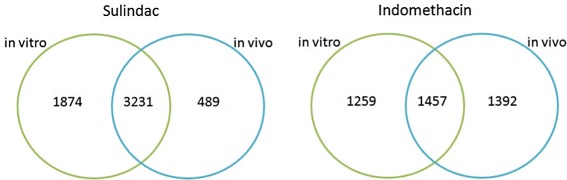
Sulindac and indomethacin compounds. Number of genes with significant dose-response relationships in *in vitro* data only, in *in vivo* data only and in both data simultaneously

The data were analysed in two ways depending on the direction of the projected fractional polynomial models. The first set of models (*in vitro disconnects*) defined the fractional polynomial powers based on the *in vitro* data set and projected its dose-response profiles to the *in vivo* data set. The second set of models (*in vivo disconnects*) defined the fractional polynomial powers based on the *in vivo* data set and projected its dose-response profiles to the *in vitro* data set. The resulting number of in vitro and in vivo disconnects for Sulindac and Indomethacin are illustrated in Fig. 4. The analyses were performed in statistical software R version 3.0.1 [39]. The R scripts are available upon request from the authors.

### *In vitro* disconnects

The number of the identified disconnected genes per compound ranged from 0 to 1,276. Three genes (*Aldh1a1*, *Cyp1a1* and *Fam25a*) were consistently identified in 56 compounds whilst 446 genes were detected in more than 10 compounds. The 446 genes were analysed further for common biological pathways using GO [40], KEGG [41] and *Janssen pharmaceutica* in-house gene databases. As expected, many of the genes are involved in drug metabolism (e.g. acetaminophen metabolism, Benzo[a]pyrene metabolism, CAR/RXR activation, PXR/RXR activation), as well as endogenous compound metabolism (e.g. butanoate metabolism, alanine, cysteine and methionine metabolism, nitrogen metabolism, fatty acid metabolism, cholesterol biosynthesis). Additionally, some of the genes are also involved in toxicity related pathways such as oxidative stress due to reactive metabolites, bilirubin increase, glutathion depletion and phospholipidosis as well as complex pathways such as immune response, classical complement and coagulation. Only pathways containing more than five genes and with coverage of more than 10 *%* (i.e., more than 10 *%* of their genes were disconnected genes) were considered.

**Fig. 5:**
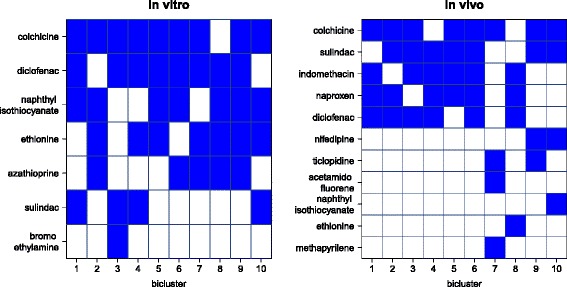
Biclustering results. Appearance of compounds across 10 biclusters. The blue colour indicates membership of a bicluster. Left panel: An example of *in vitro disconnects*. Right panel: An example of *in vivo disconnects*

We further identified 188 unique genes that were consistently defined as disconnected genes in seven compounds based on the first 10 biclusters from the Bimax algorithm (left panel on Fig. 5). Sulindac and diclofenac are both anti-inflammatory drugs, acetic acid derivatives that are likely to damage liver [42]. Naphthyl isothiocyanate was shown to cause direct hepatotoxicity [43]. Among the 188 genes, the top genes (with respect to fold change) are associated with liver toxicity. Genes *A2m* and *Lcn2* were validated for being affected in case of hepatotoxicity [44]. Other toxicity associated genes found were *Cyp1a1*, *Pcsk9*, *Car3*, *Gstm3* or *Ccnd1*. Table 1 shows the results of pathway analysis for the first bicluster (compounds: sulindac, naphthyl isothiocyanate, diclofenac and colchicine). For complete results of biclustering *in vitro*, see Additional files 1 and 2.

**Table 1:** Examples of the clustered disconnected genes and their probable pathways. The pathways were identified using KEGG [41]. The genes are members of first bicluster of the analysis starting with *in vitro* data. The compounds in first bicluster are sulindac, naphthyl isothiocyanate, diclofenac and colchicine

Pathway	Genes
Complement and coagulation cascades	*A2m C1s C5 C8a C4bpb Cfh F5*
Chemical carcinogenesis	*Cyp1a1 Gstm3 Gsta5*
Metabolism of xenobiotics	*Akr7a3 Cyp1a1 Gstm3 Gsta5*
Pathways in cancer	*Ccnd1 Fn1 Lamc2*

### *In vivo* disconnects

There were 175 genes that showed disconnect in gene expression from *in vivo* to *in vitro* rat experiments for more than 10 compounds. Similar pathways as in the previous section (i.e projection from *in vitro* to *in vivo*) were also discovered, although more of the pathways were related to exogenous compound metabolism. Oxidative stress endpoints related pathways were more common *in vivo*. Complex pathways such as complement and coagulation identified in the *in vitro* data set were not discovered in the analysis of the *in vivo* data set, which may be due to differences between the prescribed dose and actual exposure in liver tissue *in vivo*.

The Bimax algorithm identified 163 unique genes common to 11 distinct compounds based on the first 10 biclusters (right panel on Fig. 5). Five compounds were identified both in *in vitro* and *in vivo* analyses of disconnects: sulindac, colchicine, diclofenac, ethionine and naphthyl isothiocyanate. The most interesting of the additional compounds are indomethacin and naproxen. They are both members of a group of non-steroidal anti-inflammatory drugs (NSAIDs), the former an acetic acid derivative and the latter a propionic acid derivative. Both drugs are nonselective cyclooxygenase (COX) isozyme inhibitors, i.e. with undesired targeting of COX-1 that leads to gastrointestinal adverse effects [45, 46]. Specifically, both drugs are indicated as high risk drugs for general upper gastrointestinal complications [47]. All of the compounds are connected to toxicity events. Most of the toxicity related genes (*A2m*, *Lcn2*, *Car3*, *Pcsk9*, *Acsl1*, *Lamc2*, *Selenbp1* and *Serpina10*) from the previous *in vitro* analysis were also identified from the analysis of the *in vivo* data set. Other toxicity related genes were *Cyp2e1* [48], *Upp1*, *Gss*, *Ddc*, *Gstm7* and *Srebf1*. For complete results of biclustering *in vivo*, see Additional files 3 and 4.

### Simulation study

The empirical validation of the fractional polynomial method in the context of *in vitro* and *in vivo* disconnects was done through two simulation studies. In the first simulation study, data were generated according to seven possible scenarios. First setting corresponded to the null model of no disconnect between *in vitro* and *in vivo* experiments. The other six settings corresponded to three groups of genes: genes with opposite dose-response profiles for *in vitro* and *in vivo*, genes with dose effect only for *in vivo* and dose effect only for *in vitro*. The settings followed either linear model or second order fractional polynomial model. For each setting, 10,000 data sets were generated.

According to the simulation results, the proposed projected fractional polynomial method under the null model resulted in 90 % specificity using the same number of dose and the same number of observations per dose as in TGP data set. When number of observations per dose was increased to four, specificity increased up to 98 %. Under the alternative hypothesis of a disconnected dose-response profiles between *in vitro* and *in vivo* experiments, the method resulted in 100 % sensitivity for the disconnected linear profiles. For nonlinear profiles, sensitivity of 80–95 % was achieved, for the maximum fold change between the *in vitro* and *in vivo* settings greater than 1.2. Sensitivity increased up to 98–100 % when the fold change was greater than 1.6.

The second simulation study mimicked the structure of the TGP experiment. In total, 6,000 genes were generated to create one data set. Half of them contained no dose effects for both *in vitro* and *in vivo*. The other half exhibited clear dose-response effect *in vitro* and opposite dose-response effect *in vivo*, creating a disconnect between two data sets. Specifically, the model used for *in vitro* was second order polynomial model with fold change of one (that was the minimal fold change of interest in our analysis). Standard deviation and the number of observations per dose correspond to the TGP data set. LRTs for dose-response and interaction were applied for each gene. The resulting p-values were adjusted for multiplicity using Benjamini-Hochberg procedure to control false discovery rate (BH-FDR) at 10 %. The sensitivity and specificity was computed as amount of correctly identified genes from both categories (null model and true disconnect). The whole procedure was repeated for 1,000 simulated data sets, computing sensitivity and specificity for each of them.

**Fig. 6:**
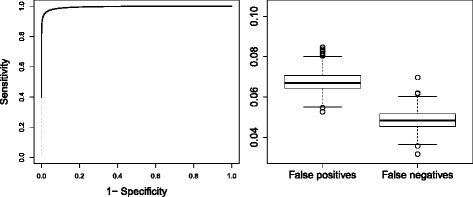
Validation of fractional polynomials: Results of simulation study. Left panel: ROC curves for sensitivity and specificity of all simulated data set (grey dotted lines) and averaged ROC curve (black solid line). Right panel: Box plot of false positives and false negatives of all 1,000 simulated data sets

The ROC curves of all the simulated data sets are shown in left panel of Fig. 6, together with the averaged ROC curve. The spread of ROC curves is very low, suggesting stability of the method across the simulated data sets. When FDR was controlled at 10 %, average specificity and sensitivity were 93 % and 95 %, respectively. The box plots of false positives and false negatives for the simulated data set are shown in Fig. 6 (right panel). The FDR is well controlled at the desired level of 10 % and false negatives rate is very low.

The simulation studies indicated that the method may perform better in other settings than the reported results for the TGP experiment due to its limited number of replicates per dose and the weak signals. The full description of the simulations settings and results can be found in the Additional file 5.

## Discussion

**Fig. 7:**
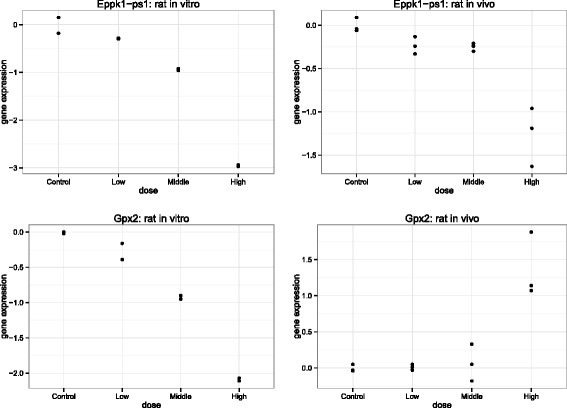
Group 1 example: compound sulindac. Two genes from Group 1. Top panels: gene *Eppk1-ps1* with the same direction of dose-response relationships, but different magnitude of fold change. Bottom panels: gene Gpx2 with different direction of dose-response relationships across platforms. Left panels: *in vitro*. Right panels: *in vivo*

**Fig. 8:**
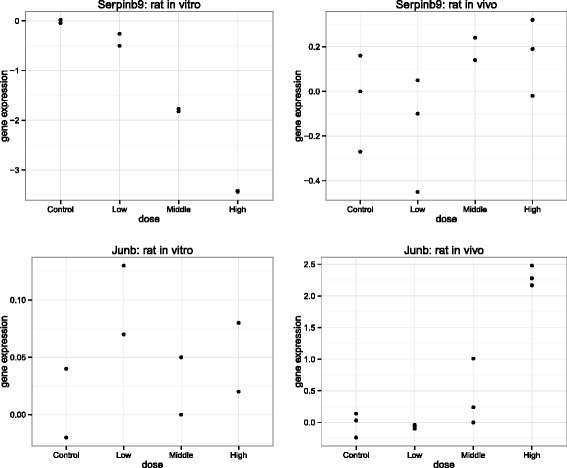
Group 2 and 3 examples: compound sulindac. Top panels: gene *Serpinb9* from Group 2, with significant dose-response relationship only in *in vitro* data. Bottom panels: gene *Junb* from Group 3, with significant dose-response relationship only in *in vivo* data. Left panels: *in vitro*. Right panels: *in vivo*

The analytic framework identified three broad groups of genes from a joint analyses of *in vitro* and *in vivo* rats toxicogenomic experiments. The first group showed a significant dose-response relationship in both the *in vitro* and *in vivo* toxicogenomic experiments. These types of genes are shown in the top panels of Fig. 7. Whilst some of the genes in this group showed contradictory dose-responses profiles between the *in vitro* and *in vivo* data, others showed similar dose-response profiles, but with different magnitude of gene expression values. The second group contains genes that showed a significant dose-response relationship in *in vitro* experiments, but not in *in vivo* experiments. Example of such genes are presented in the top panels of Fig. 8. This set of genes may represent the difference in biological complexity between *in vivo* and *in vitro* systems. The third group are those genes that showed significant dose-response relationship in *in vivo* experiments, but not in *in vitro* experiments. For complete results, see Additional files 6 and 7.

This set of genes may occur due to the mechanism of action (MoA) *in vitro* of a drug candidate not being representative of *in vivo*. Most of the compounds in our specific case study that triggered the expression of the identified disconnected genes are members of a group of anti-inflammatory drugs and all of them are related to hepatotoxicity, nephrotoxicity or gastro-intestinal toxicity.

If additional data about experiments are available both for *in vitro* and *in vivo*, such data can be included in the proposed methodology. The adjustment can be done by adding the new variables in the fractional polynomial model as covariates. Note that in this type of gene expression studies, the rats are specially bred to ensure baseline comparability across all rats.

## Conclusion

The findings demonstrated that substantial differences may be identified between *in vitro* and *in vivo* gene expression. While this result is not surprising, the importance of the analysis is in the identification of different groups of the disconnected genes. Genes were identified that showed significant dose-response relationships with compounds *in vitro* that were absent *in vivo*, and vice-versa. Moreover, there was a group of genes with a different direction of dose-response relationship across the two systems. These finding confirms possibility of important discrepancies between *in vitro* experiments and *in vivo* experiments. Pathway analysis of the identifying disconnected genes between *in vivo* and *in vitro* rat system may improve our understanding of uncertainties in mechanism of action of a drug candidate in human, especially for early toxicity detection.

## Availability of supporting data

The data sets supporting the results of this article are available in the TG-GATEs Toxicogenomics Project repository (http://toxico.nibio.go.jp/english/index.html).

## Ethical approval

There was no ethical approval needed for this manuscript, because it was based on publicly available data sets. The ethical approval for the original TGP study was granted by the Ethics Review Committee for Animal Experimentation of the National Institute of Health Sciences, Japan, and by the respective contract research organizations [49].

## Additional files

Additional file 1
**Genes of top 10 biclusters** ***in vitro*** **.** List of disconnected genes that appeared in the top 10 biclusters *in vitro*.

Additional file 2
**Top 10 biclusters** ***in vitro*** **.** List of top 10 biclusters *in vitro* with all the disconnected genes and compounds that are bicluster members.

Additional file 3
**Genes of top 10 biclusters** ***in vivo*** **.** List of disconnected genes that appeared in the top 10 biclusters *in vivo*.

Additional file 4
**Top 10 biclusters** ***in vivo*** **.** List of top 10 biclusters *in vivo* with all the disconnected genes and compounds that are bicluster members.

Additional file 5
**Supplementary appendix.** Additional figures on gene expression profiles. Simulation study based validation of the methodology.

Additional file 6
**Integrated results - sulindac.** List of disconnected genes for sulindac organized in three groups (see Discussion).

Additional file 7
**Integrated results - all.** List of disconnected genes for all compounds organized in three groups (see Discussion).

## Abbreviations

TG-GATEs: Toxicogenomics Project - Genomics assisted toxicity evaluation system
TGP: The Toxicogenomics Project
I/NI: Informative/non-informative
AIC: Akaike’s information criterion
LRT: Likelihood ratio test
GO: The gene ontology consortium
KEGG: Kyoto encyclopedia of genes and genomes
