# Supplementary material for: Identification of in vitro and in vivo disconnects using transcriptomic data
Source: BMC Genomics. 2015 Aug 18;16(1):615. doi: 10.1186/s12864-015-1726-7 (PMC4539666; doi:10.1186/s12864-015-1726-7)
Supplement: Additional file 5 — Supplementary appendix. Additional figures on gene expression profiles. Simulation study based validation of the methodology. [file 12864_2015_1726_MOESM5_ESM.pdf]

Identification of *in vitro* and *in vivo* disconnects using transcriptomics data
Supplementary appendix

Martin Otava et al.

1 Introduction

This document contains additional materials that were not presented in the manuscript and provides detailed information about the methodology. Section 2 describes the settings and interpret the results of simulation studies that were conducted in order to validate the fractional polynomial methodology in context of interest. In Section 3, examples of the genes found as disconnected are visualized and the possible relationships of profiles between *in vitro* and *in vivo* data set are discussed.

2 Simulation study: validation of fractional polynomial method

In order to evaluate a performance of the proposed modelling approach, we estimate a sensitivity and specificity. The two quantities are defined as follows. The specificity represents the rate of genes with no disconnect that are correctly not identified as disconnected genes (i.e. related to Type I error). The sensitivity represents the rate of truly disconnected genes being identified as disconnected (i.e. power of the method). The closer to one both quantities are, the better is the performance of the method. Two simulation studies were conducted in order to validate the performance of the suggested methodology. The first simulation study was focused on evaluation of sensitivity and specificity on the single gene expression experiment. The second study generated the data set resembling the structure of the data in the TGP and focused on the multiplicity adjustment, i.e. testing on thousands of genes simultaneously.

2.1 Simulation study I: Performance of proposed method

2.1.1 Simulation settings

In the first simulation study, data were generated according to seven possible scenarios. The first setting (A in Table 1) corresponds to the null model of no disconnect between two data sets. The mean profile of the other settings are presented in Table 1 and shown in Figure 1

(for choice $Q = 1.5$). They are generated either under a linear model (B, C, D) or a second order fractional polynomial (B2, C2, D2). The settings correspond to three groups described in the Discussion Section of main manuscript: genes with opposite direction of effect of the dose for *in vitro* and *in vivo* data (B, B2), genes with dose effect only for *in vivo* data (C, C2) and dose effect only for *in vitro* data (D, D2). For each setting, $N = 10,000$ data sets were generated.

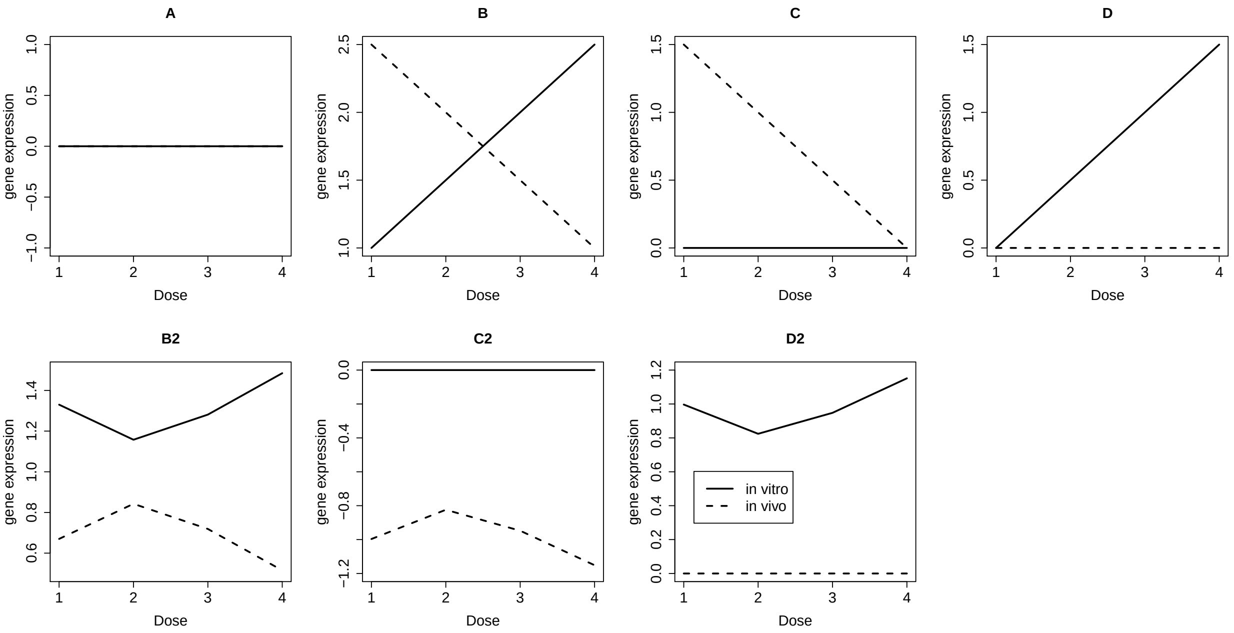

Figure 1: The profiles used in the simulation study: means used for *in vitro* (solid line) and *in vivo* (dashed line) for the four simulation scenarios. In scenario 'A', both profiles overlap each other.

Polynomial	Setting	Model *in vitro*	Parameters *in vitro*	Model *in vivo*	Parameters *in vivo*
Null model	A	$Y_{ij} = 0 + \varepsilon_{ij}$	$\beta_0 = \beta_1 = \beta_2 = 0$	$Y_{ij} = 0 + \varepsilon_{ij}$	$\beta_0 = \beta_1 = \beta_2 = 0$
Linear	B	$Y_{ij} = (1 - \frac{Q}{3}) + \frac{Q}{3}D + \varepsilon_{ij}$	$\beta_0 = 1 - \frac{Q}{3},\ \beta_1 = \frac{Q}{3},\ \beta_2 = 0,\ p_1 = 1$	$Y_{ij} = (1 + \frac{4Q}{3}) - \frac{Q}{3}D + \varepsilon_{ij}$	$\beta_0 = 1 + \frac{4Q}{3},\ \beta_1 = -\frac{Q}{3},\ \beta_2 = 0,\ p_1 = 1$
	C	$Y_{ij} = 0 + \varepsilon_{ij}$	$\beta_0 = \beta_1 = \beta_2 = 0$	$Y_{ij} = \frac{4Q}{3} - \frac{Q}{3}D + \varepsilon_{ij}$	$\beta_0 = \frac{4Q}{3},\ \beta_1 = -\frac{Q}{3},\ \beta_2 = 0,\ p_1 = 1$
	D	$Y_{ij} = 0 - \frac{Q}{3} + \frac{Q}{3}D + \varepsilon_{ij}$	$\beta_0 = -\frac{Q}{3},\ \beta_1 = \frac{Q}{3},\ \beta_2 = 0,\ p_1 = 1$	$Y_{ij} = 0 + \varepsilon_{ij}$	$\beta_0 = \beta_1 = \beta_2 = 0$
2nd order	B2	$Y_{ij} = 1 + \frac{Q}{50}D^2 + \frac{Q}{5}D^{-3} + \varepsilon_{ij}$	$\beta_0 = 1,\ \beta_1 = \frac{Q}{50},\ \beta_2 = \frac{Q}{5},\ p_1 = 2,\ p_2 = -3$	$Y_{ij} = 1 - \frac{Q}{50}D^2 - \frac{Q}{5}D^{-3} + \varepsilon_{ij}$	$\beta_0 = 1,\ \beta_1 = -\frac{Q}{50},\ \beta_2 = -\frac{Q}{5},\ p_1 = 2,\ p_2 = -3$
	C2	$Y_{ij} = 0 + \varepsilon_{ij}$	$\beta_0 = \beta_1 = \beta_2 = 0$	$Y_{ij} = -\frac{2}{3} - \frac{Q}{50}D^2 - \frac{Q}{5}D^{-3} + \varepsilon_{ij}$	$\beta_0 = -\frac{2}{3},\ \beta_1 = -\frac{Q}{50},\ \beta_2 = -\frac{Q}{5},\ p_1 = 2,\ p_2 = -3$
	D2	$Y_{ij} = \frac{2}{3} + \frac{Q}{50}D^2 + \frac{Q}{5}D^{-3} + \varepsilon_{ij}$	$\beta_0 = \frac{2}{3},\ \beta_1 = \frac{Q}{50},\ \beta_2 = \frac{Q}{5},\ p_1 = 2,\ p_2 = -3$	$Y_{ij} = 0 + \varepsilon_{ij}$	$\beta_0 = \beta_1 = \beta_2 = 0$

Table 1: Simulation settings. The first two columns determine the type of profile and identification of the setting. Following two columns states explicitly the model used for particular setting for *in vitro* and the values of parameters in model (1) of main manuscript (for case of $p_1 \neq p_2$). Last two columns shows the same for *in vivo*. The specification of parameters p_1, p_2 is omitted if $\beta_1 = 0$ or $\beta_2 = 0$, respectively.

For setting A, the data were generated under varying noise, i.e. with $\varepsilon_{ij} \sim N(0, SD^2)$, where $SD = 0.01, 0.14, 0.25, 0.5, 1, 1.5$. Additionally, the data were generated twice, once with same amount of observations per dose as original TGP data (two for *in vitro* and three for *in vivo*) and once with four observations per dose in both data sets.

All the remaining settings (B, C, D, B2, C2, D2) were generated with value of $Q = 1.5, 2, 3$ and $\varepsilon_{ij} \sim N(0, 0.14^2)$. For settings B, C, D, the constant Q equals the fold change (as defined in the manuscript, i.e. maximal difference of dose-specific means between the two data sets). The actual fold change for settings B2, C2 and D2 resulting from values of Q is given in Table 4 below. The standard deviation was used as $SD = 0.14$ which approximately correspond to 75% quantile of all the variances across all he compound, both for *in vitro* and *in vivo* data. The same number of observations as in the original TGP data set were used.

When the data were analysed, both test for dose-response and test for interaction were applied with level of significance 0.1. For all the settings was conducted analysis starting with *in vitro* data set, except for settings C and C2, where analysis starting from *in vivo* data set was conducted (otherwise, no disconnect would be detected, because there is no signal for *in vitro* data in C and C2).

The results for sensitivity and specificity for all scenarios are shown in Table 2, Table 3 and Table 4, respectively. The specificity of separate LRTs (Table 2) is lower than value 0.9. It is caused by the AIC procedure that fits selects the best powers. The small amount of observations, especially for *in vitro* data, causes fitting more complex models than necessary. However, we can see that using both tests together (column 'Disconnect') corrects specificity of disconnect determination (given the 0.1 significance level used for testing). Additionally, a small increase of observations number per dose to $n = 4$ would improve the performance of individual tests.

The high sensitivity for LRT in case of linear model is apparent for any setting (Table 3). The effect of fold change of one (that was considered as lowest important in our analysis) is found in all $N = 10,000$ simulated data sets. Similar pattern can be detected, when data were generated according to second order fractional polynomial models (Table 4). The detection of disconnect is driven by dose-response detection mainly, because interaction is easily detected in all the settings. For all the settings, we can see high sensitivity for the values close to fold change of one which was the lowest effect of interest in our analysis and

approaching maximal possible sensitivity already at fold change less than two. The higher sensitivity in setting C2 compared to D2, while having same fold change, occurs due to the dose-response effect estimated using three observations per dose *in vivo* instead of only two for *in vitro* data set. The same sensitivity for model B2 and C2 is given by fact that their dose-response profile *in vitro* is parallel, i.e. the LRT tests the same mean structure.

Table 2: Specificity of the methodology for single experiment. The first columns determine the type of profile, number of observations per dose and the value of SD that was used to generate noise. For number of observations, TGP denotes same setting as in original data set and $n = 4$ four observations for both data sets. Following three columns show specificity of LRTs. Third column shows specificity of LRT for significance of dose-response relationship *in vitro*. Fourth column shows specificity of LRT for significance of interaction, i.e. projection of optimal fractional to both data sets. Last column represents test for disconnect, i.e. gene being significant in both LRTs for dose-response and interaction. All tests use significance level 0.1. Results of each row are based on mean of 10,000 experiments.

Profile	n	SD	*in vitro* dose-response	Projection of FP	Disconnect
A	TGP	0.01	0.8	0.81	0.9
		0.14	0.8	0.81	0.9
		0.25	0.8	0.81	0.9
		0.50	0.8	0.81	0.9
		1.00	0.8	0.81	0.9
		1.50	0.8	0.81	0.9
	$n = 4$	0.01	0.85	0.93	0.98
		0.14	0.85	0.93	0.98
		0.25	0.85	0.93	0.98
		0.50	0.85	0.93	0.98
		1.00	0.85	0.93	0.98
		1.50	0.85	0.93	0.98

Table 3: Sensitivity of the methodology for single experiment with underlying linear model. The first two columns determine the type of profile and true underlying effect. Following three columns show sensitivity of LRTs. Third column shows sensitivity of LRT for significance of dose-response relationship *in vitro* (B, D) or *in vivo* (C). Fourth column shows sensitivity of LRT for significance of interaction, i.e. projection of optimal fractional to both data sets. Last column represents test for disconnect, i.e. gene being significant in both LRTs for dose-response and interaction. All tests use significance level 0.1. Results of each row are based on mean of 10,000 experiments.

Profile	Fold change	Dose-response	Projection of FP	Disconnect
B	0.75	0.995	1.000	0.995
	1.00	1.000	1.000	1.000
	1.50	1.000	1.000	1.000
C	0.75	1.000	1.000	1.000
	1.00	1.000	1.000	1.000
	1.50	1.000	1.000	1.000
D	0.75	0.995	1.000	0.995
	1.00	1.000	1.000	1.000
	1.50	1.000	1.000	1.000

Table 4: Sensitivity of the methodology for single experiment with an underlying second order fractional polynomial model. The first two columns determine the type of profile and true underlying effect. Following three columns show sensitivity of LRTs. Third column shows sensitivity of LRT for significance of dose-response relationship *in vitro* (B2, D2) or *in vivo* (C2). Fourth column shows sensitivity of LRT for significance of interaction, i.e. projection of optimal fractional to both data sets. Last column represents test for disconnect, i.e. gene being significant in both LRTs for dose-response and interaction. All tests use significance level 0.1. Results of each row are based on mean of 10,000 experiments.

Profile	Q	Fold change	Dose-response	Projection of FP	Disconnect
B2	1.50	0.969	0.609	1.000	0.609
	2.00	1.293	0.796	1.000	0.796
	3.00	1.939	0.976	1.000	0.976
C2	1.50	1.151	0.802	1.000	0.802
	2.00	1.313	0.951	1.000	0.951
	3.00	1.636	0.999	1.000	0.999
D2	1.50	1.151	0.609	1.000	0.609
	2.00	1.313	0.796	1.000	0.796
	3.00	1.636	0.976	1.000	0.976

2.2 Simulation study II: Multiplicity adjustment

The second simulation study mimics the structure of the TGP experiment. In total, $M = 6,000$ genes were generated to create one data set. Half of them followed the null model for both *in vitro* and *in vivo*. The other half exhibits clear dose-response effect *in vitro* and disconnect between *in vitro* and *in vivo*. Specifically, the model used for *in vitro* was second order polynomial model

$$Y_{ij} = \frac{2}{25}D^2 + \frac{2}{5}D^{-3} + \varepsilon_{ij}.$$

The same model was used *in vivo*, disconnect was caused by increasing mean in second dose by one and decreasing mean in last dose by 0.5. The mean profile of the setting is displayed in left panel of Figure 2. Such setting induce the fold change of one that was the minimal fold change of interest in our analysis. The $SD = 0.14$ was used, as in previous study, and the number of observations per dose was same as in TGP data set. Within whole data set of M genes, LRTs for dose-response and interaction were applied for each gene. The resulting p-values were adjusted for multiplicity using Benjamini-Hochberg procedure to control false discovery rate (BH-FDR). The disconnect of the gene was determined based on significance in both of the LRTs, with level of significance 0.1 used. The sensitivity and specificity was computed as amount of correctly identified genes from both categories (null model and true disconnect). The whole procedure was repeated for $N_2 = 1,000$ simulated data sets, computing sensitivity and specificity for each of them.

ROC curve of one data set is shown in middle panel of Figure 2, showing how the sensitivity and specificity changes if significance level varies. For all $N_2 = 1,000$ simulated data sets, average sensitivity and specificity were and 0.951 and 0.932, respectively. Minimal values across all $1,000$ data sets were 0.930 for sensitivity and 0.915 for specificity, suggesting consistently very good behaviour of the method when multiplicity adjustment applied. The boxplot of all the values of sensitivity and specificity for $1,000$ simulated data set is shown in right panel of Figure 2. The specificity is well controlled, always above value of 0.9 while sensitivity was still maintained very high.

In summary, both simulation studies suggest very good behaviour of the method with high sensitivity and specificity for effect of interest (fold change more than one).

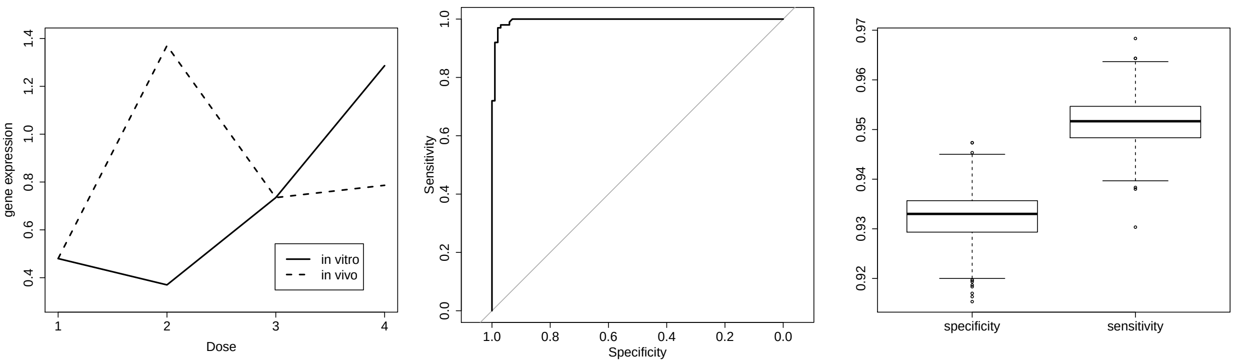

Figure 2: Left panel: The profile used in the second simulation study: means used for *in vitro* (solid line) and *in vivo* (dashed line). Middle panel: Sensitivity and specificity of one of the data sets when varying the significance level threshold. Right panel: Boxplot of sensitivity and specificity of all 1,000 simulated data sets.

3 Examples of genes

This section displays examples of profiles of genes that exhibit various types of dose-response relationship and various type of disconnect between the *in vitro* and the *in vivo* data. The first set of figures visualizes diverse genes within compound diclofenac. Figures 3, 4 and 5 show genes that were disconnected based on both directions of analysis (called 'first group' in the Discussion of main manuscript). Figures 6, 7 and 8 show further examples of genes that were found only when starting from *in vitro* data set (second group). The disconnect exhibited by these genes translates into significant dose-response relationship in the *in vitro* data set, but non-significant in the *in vivo* data set. Figures 9, 10 and 11 show genes only identified when starting from *in vivo* data set (third group). Analogously, these genes had significant dose-response relationship in the *in vivo* data set, but not in the *in vitro* data set.

The influence of the selection step is demonstrated in Figures 12, 13 and 14. Visualized genes show significant dose-response effect and interaction for *in vitro* starting analysis, but were not selected as disconnected. None of the differences between *in vitro* and *in vivo* dose-specific means was higher than necessary threshold (fold change of 1). The disconnect of such genes, even if significant, is small with respect to fold change and therefore not of our interest. A significant result can be caused by small variability within gene, not by the magnitude of effect, which is an important criterion in exploratory studies.

The nonsignificant genes (for diclofenac) are visualized in third set of figures. Figures 15, 16 and 17 present genes that do not show any dose-response relationship in any data set. Figures 18, 19 and 20 show the genes that exhibit similar dose response relationship for both data sets, so the LRT for interaction does not conclude significance of interaction compared to simpler model. Those are actually genes that seem to be translatable across platforms (although some additional appropriate analysis would have to be conducted to confirm it, which is out of the scope of presented manuscript).

The last set visualizes some of the genes contained in the first bicluster found for *in vitro* analysis. The genes are plotted for all the compounds in the bicluster: diclofenac, sulindac, naphthyl isothiocyanat and colchicine. Figures 21, 22 and 23 visualize the *in vitro* dose-response relationship for three genes found in first bicluster for all four compound that were members of that bicluster. The figures demonstrate that such genes do not need to exhibit similar relationships, because the biclustering is based only on the information about disconnect and it is not related to the shape of dose-response relationship. Interestingly, we could see that the genes tend to behave in similar way across three out of four compounds: diclofenac, sulindac and naphthyl isothiocyanate. The disconnects between *in vitro* data and *in vivo* data for the genes in first bicluster are shown in the Figures 24, 25 and 26. Again, it can be seen that the type of disconnect can differ within one bicluster due to the fact that biclustering was conducted only on the level of information if there was disconnect or not and did not depend on the shape of dose-response profiles.

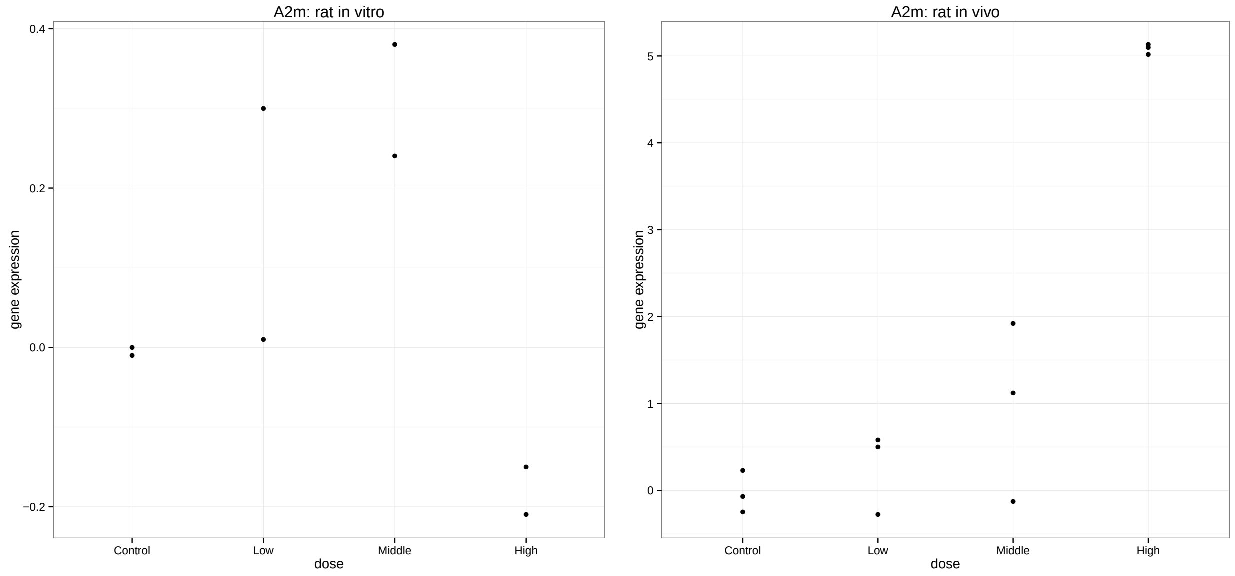

Figure 3: Compound diclofenac and gene *A2m*. Left panel: *in vitro*. Right panel: *in vivo*.

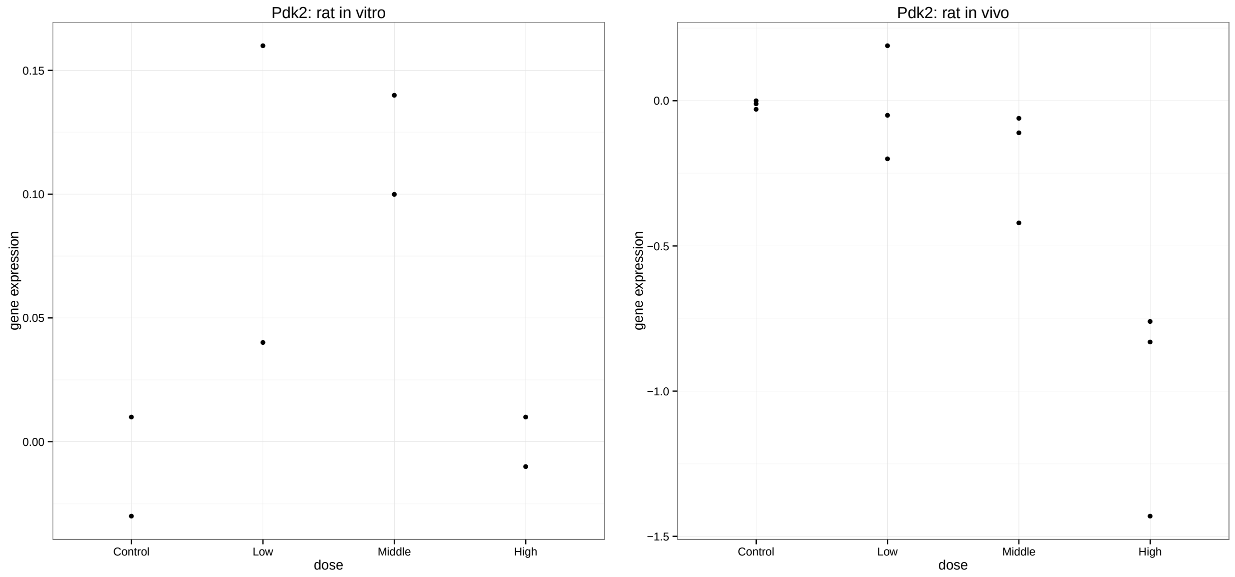

Figure 4: Compound diclofenac and gene *Pdk2*. Left panel: *in vitro*. Right panel: *in vivo*.

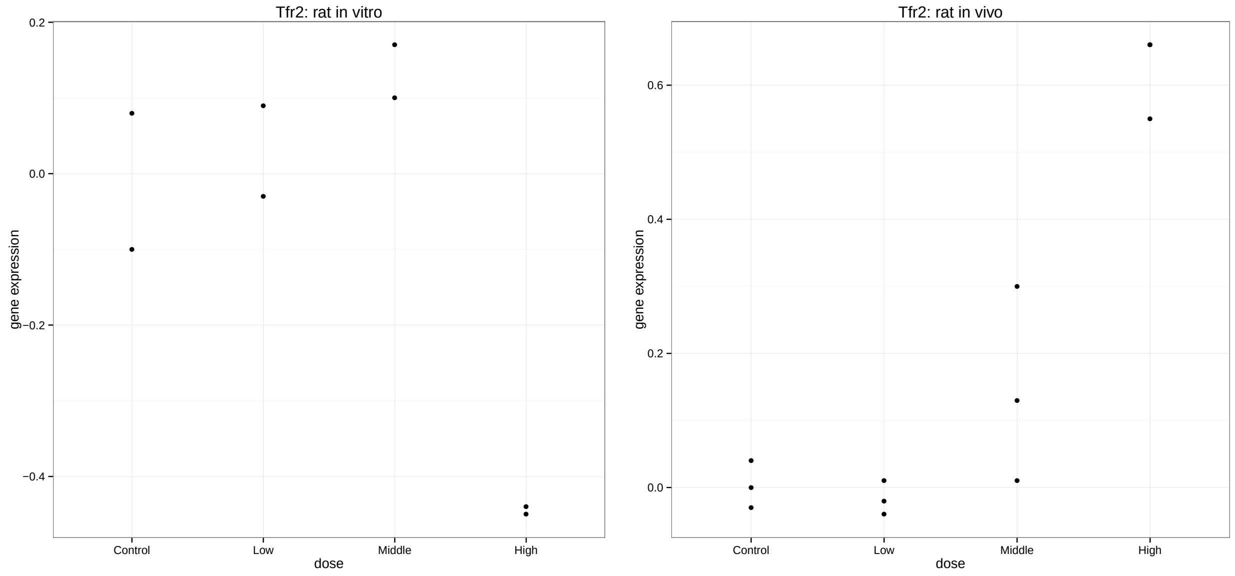

Figure 5: Compound diclofenac and gene *Tfr2*. Left panel: *in vitro*. Right panel: *in vivo*.

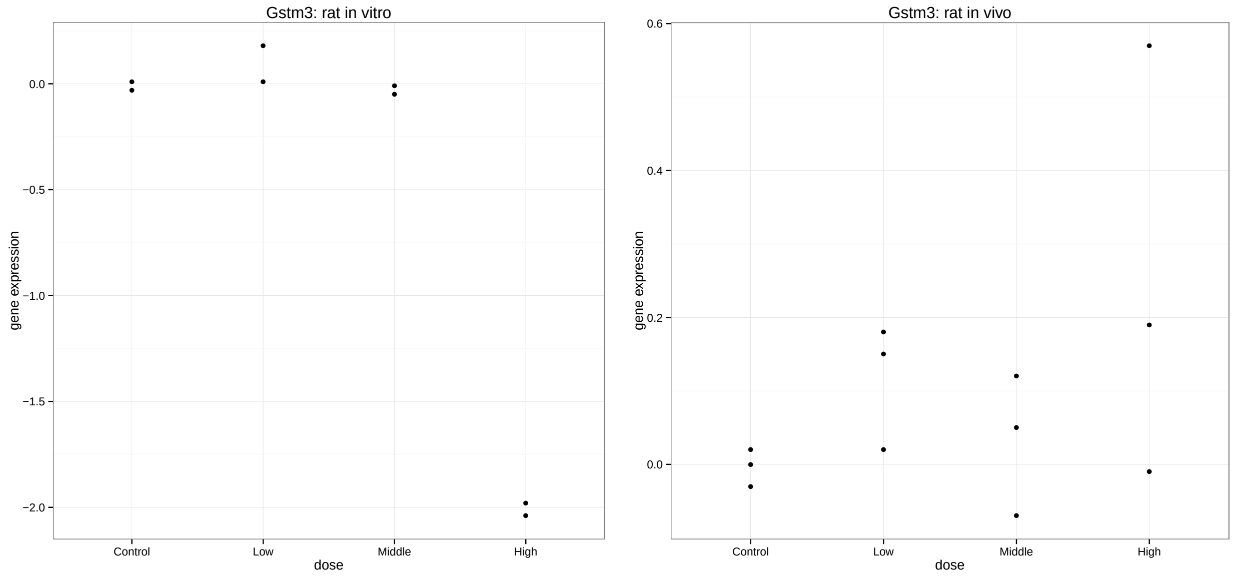

Figure 6: Compound diclofenac and gene *Gstm3*. Left panel: *in vitro*. Right panel: *in vivo*.

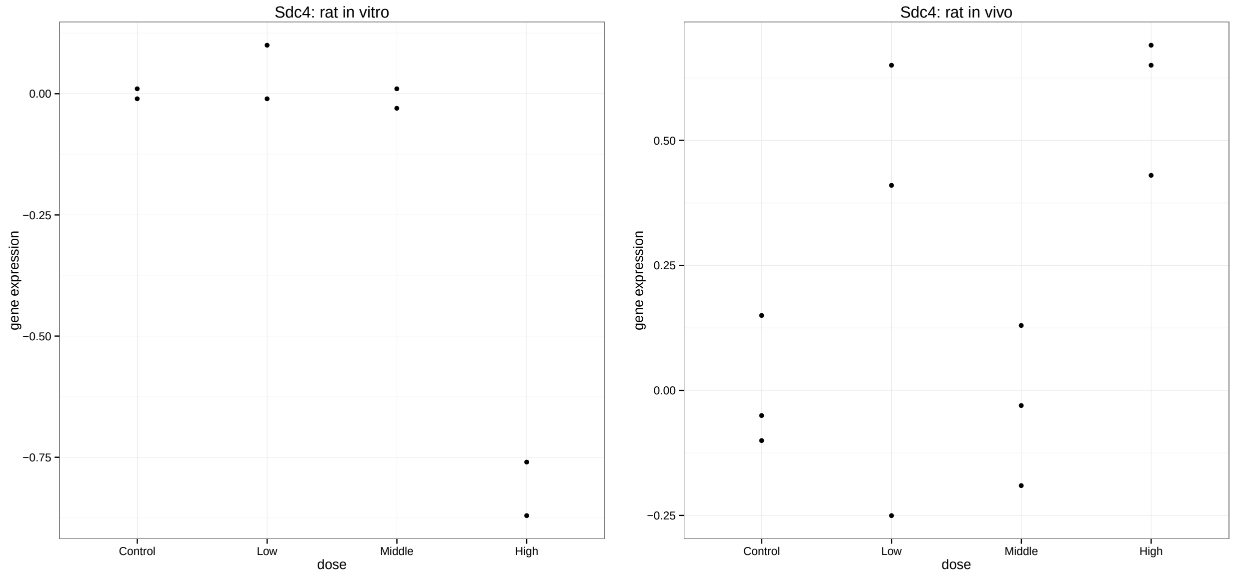

Figure 7: Compound diclofenac and gene *Sdc4*. Left panel: *in vitro*. Right panel: *in vivo*.

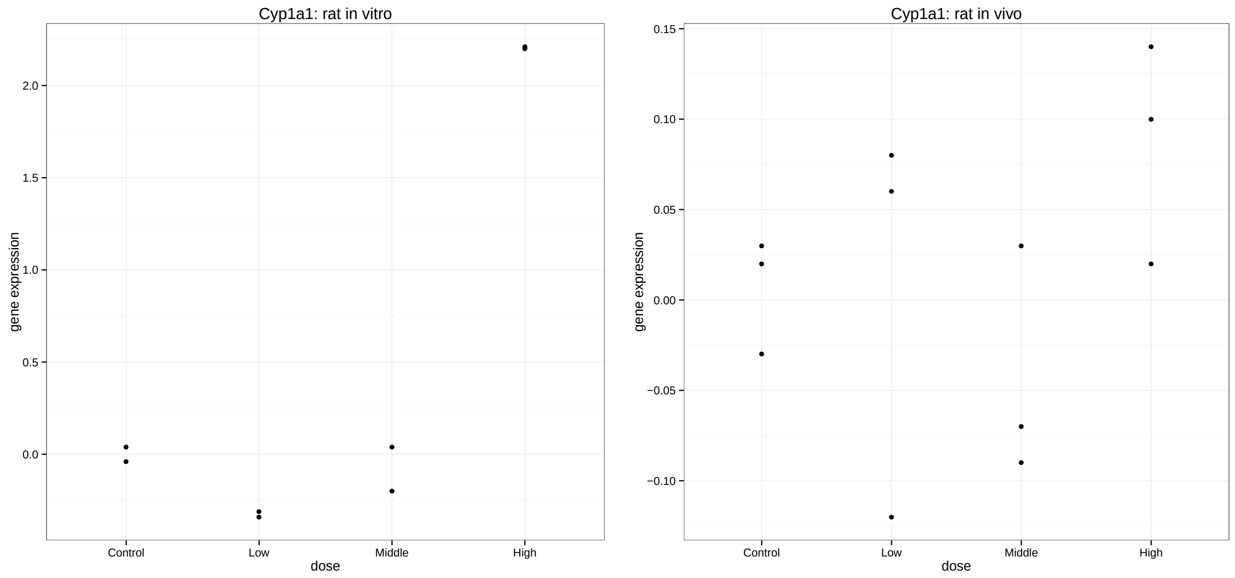

Figure 8: Compound diclofenac and gene *Cyp1a1*. Left panel: *in vitro*. Right panel: *in vivo*.

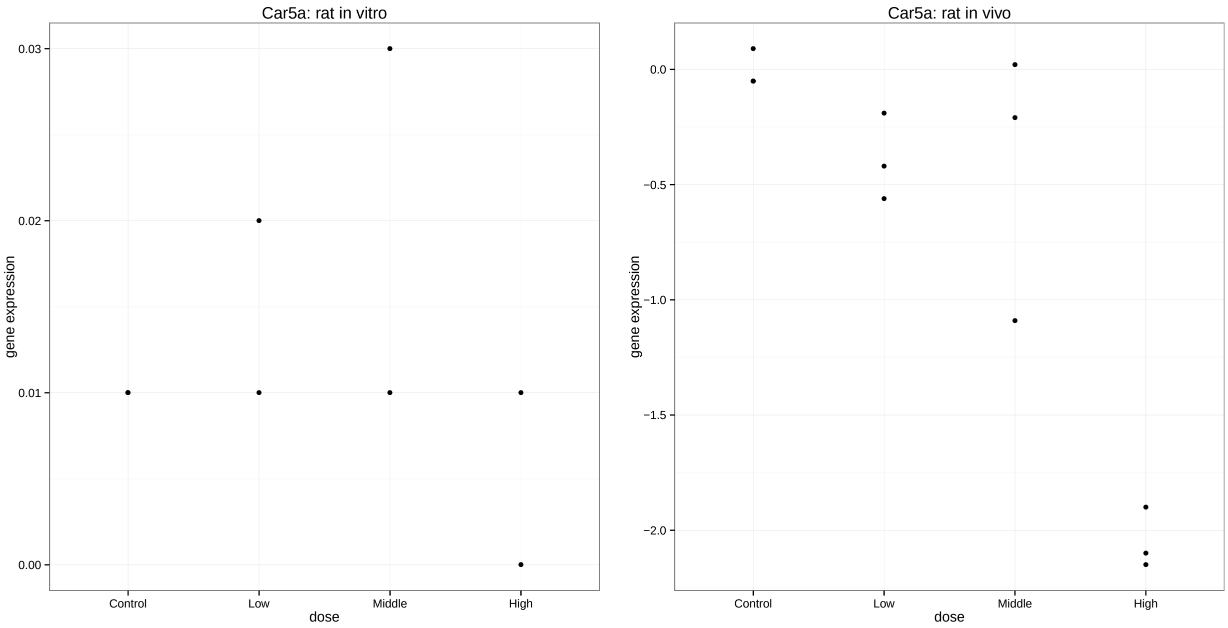

Figure 9: Compound diclofenac and gene *Car5a*. Left panel: *in vitro*. Right panel: *in vivo*.

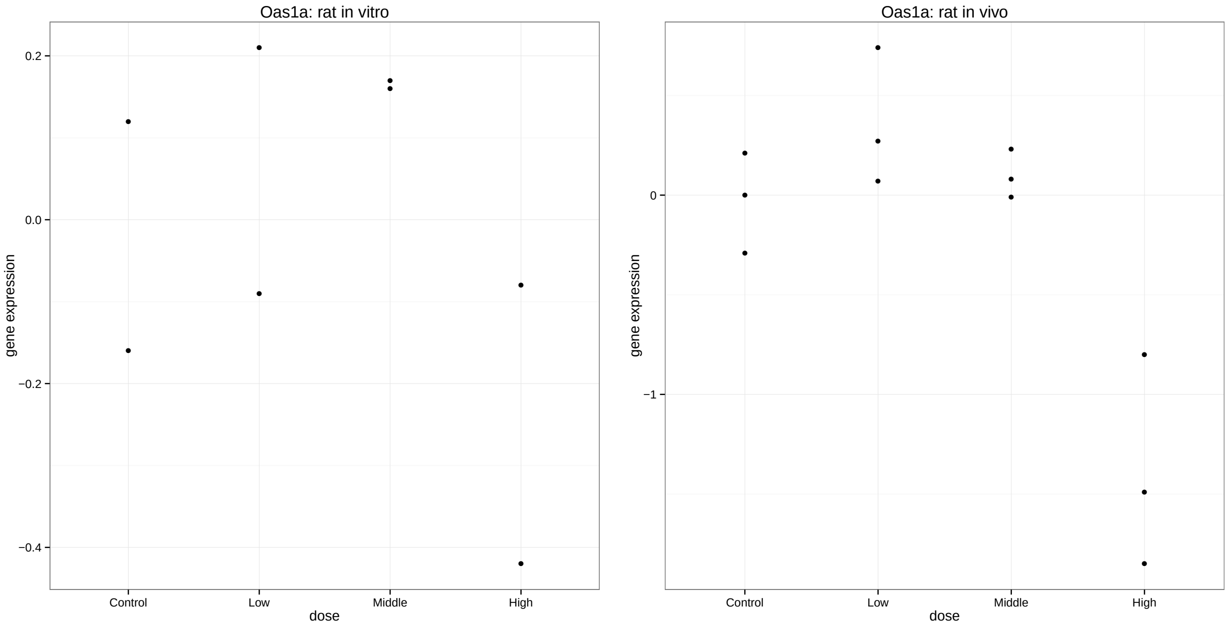

Figure 10: Compound diclofenac and gene *Oas1a*. Left panel: *in vitro*. Right panel: *in vivo*.

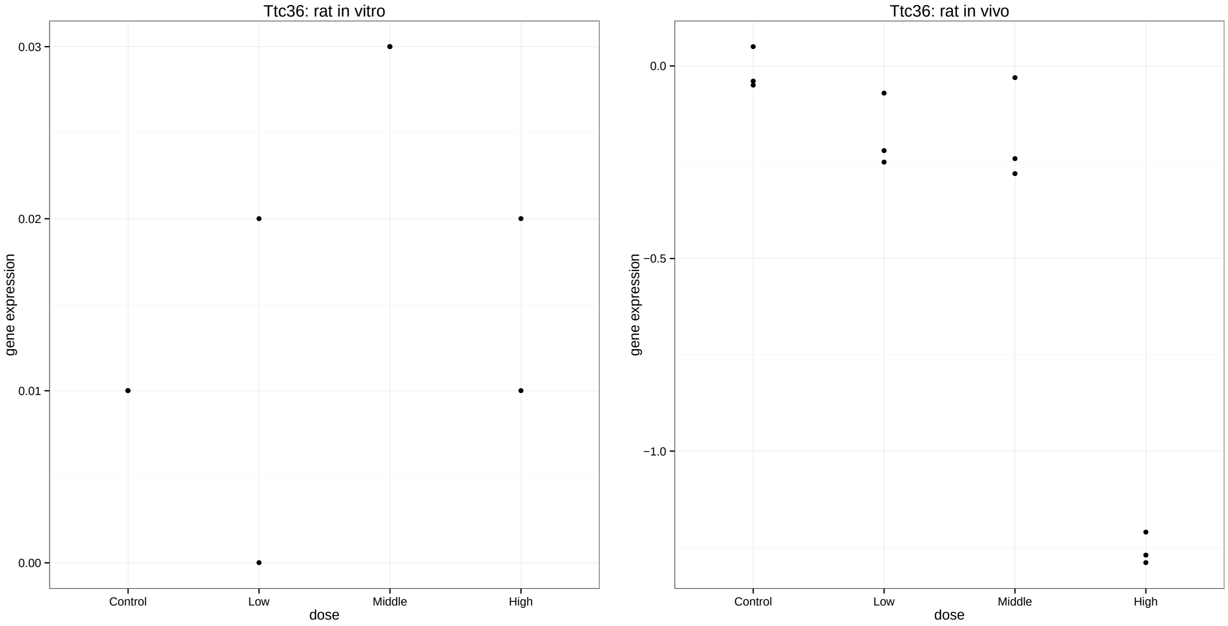

Figure 11: Compound diclofenac and gene *Ttc36*. Left panel: *in vitro*. Right panel: *in vivo*.

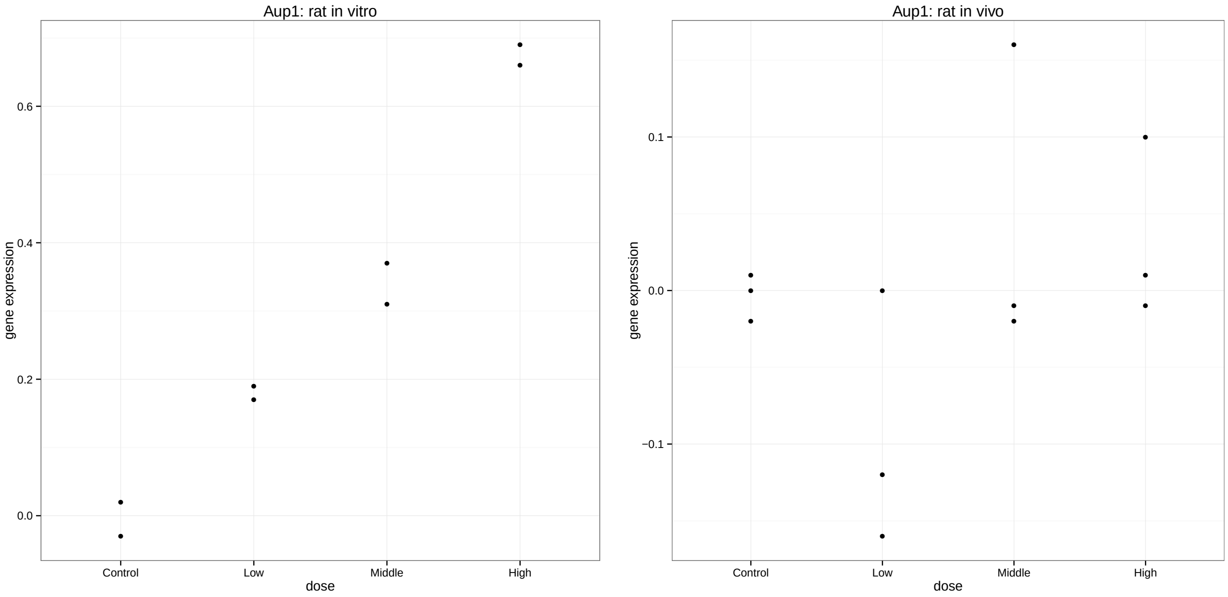

Figure 12: Compound diclofenac and gene *Aup1*. Left panel: *in vitro*. Right panel: *in vivo*.

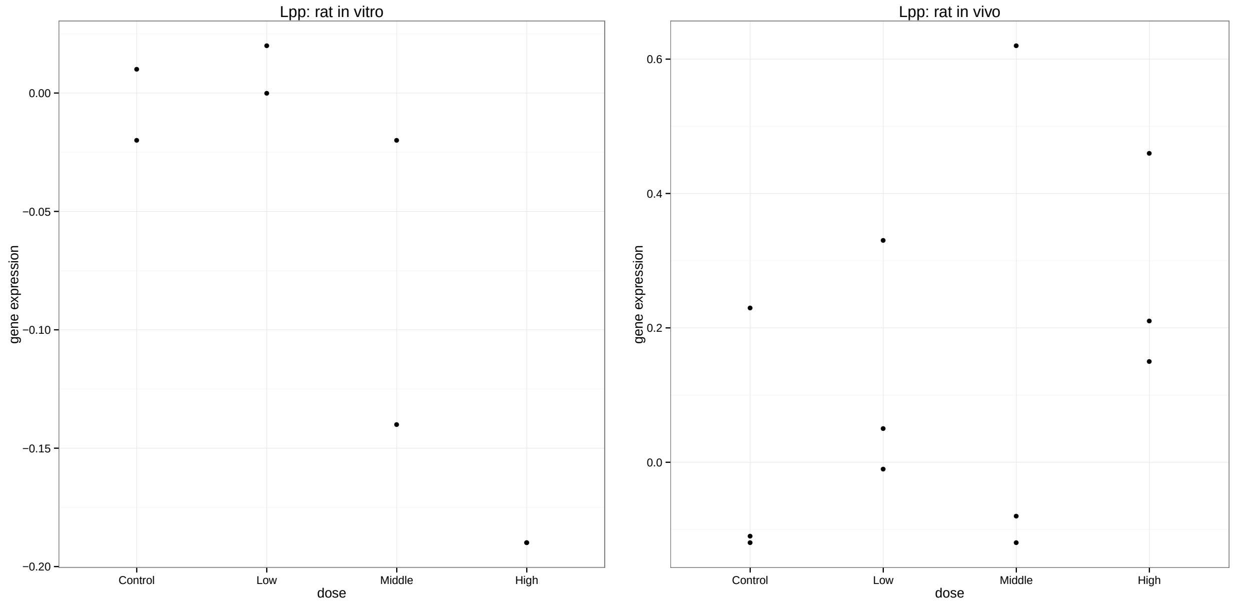

Figure 13: Compound diclofenac and gene *Lpp*. Left panel: *in vitro*. Right panel: *in vivo*.

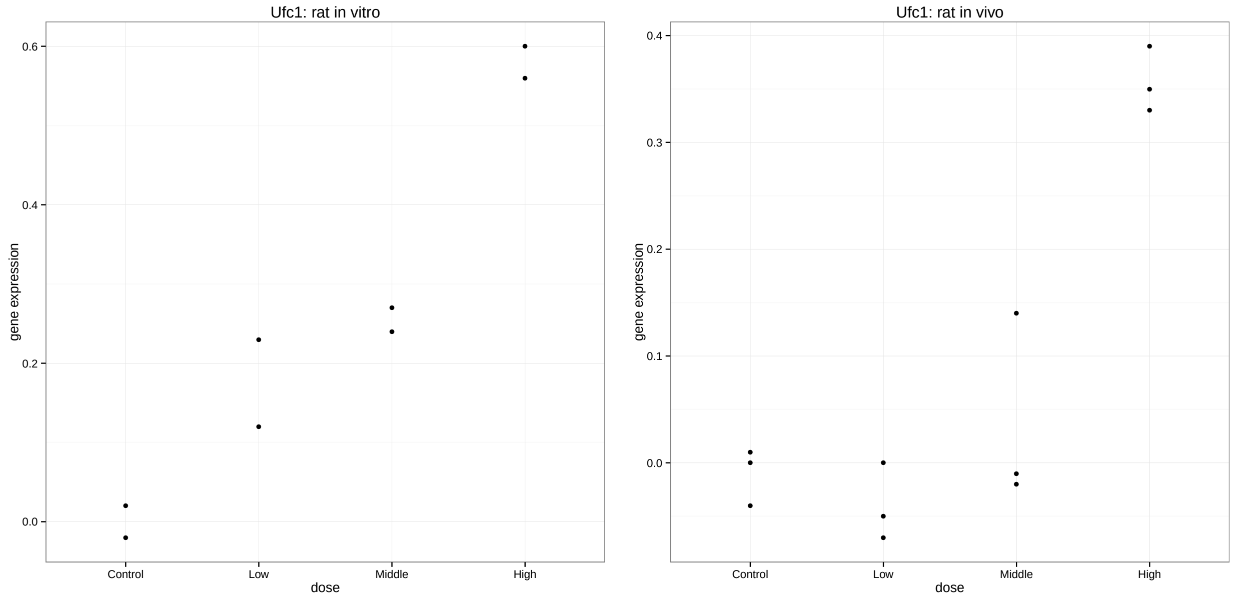

Figure 14: Compound diclofenac and gene *Ufc1*. Left panel: *in vitro*. Right panel: *in vivo*.

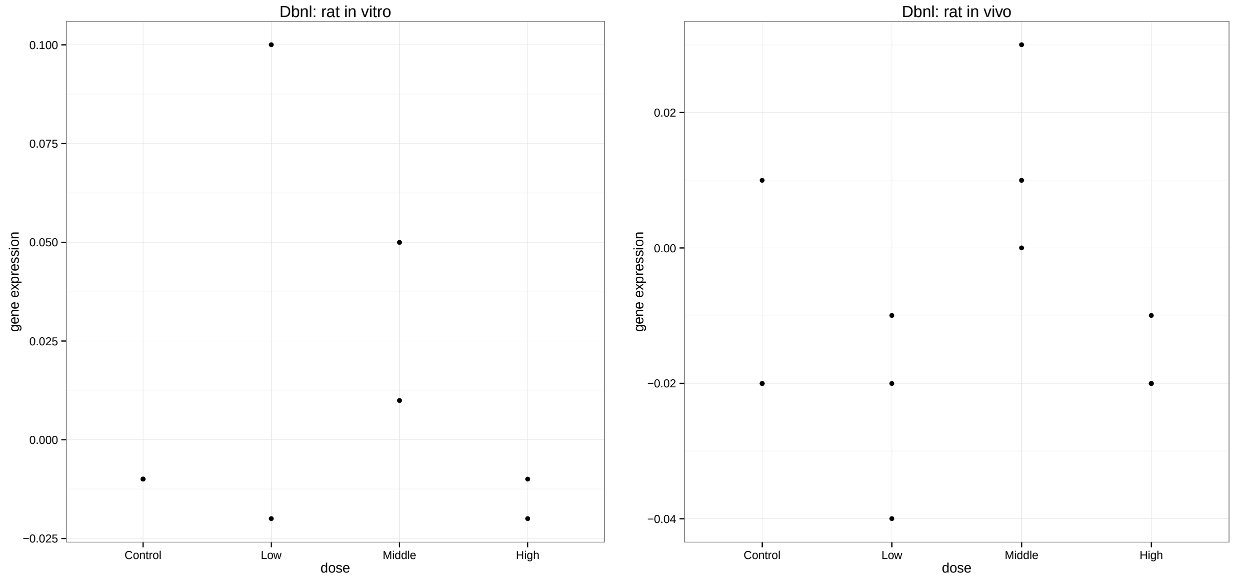

Figure 15: Compound diclofenac and gene *Dbnl*. Left panel: *in vitro*. Right panel: *in vivo*.

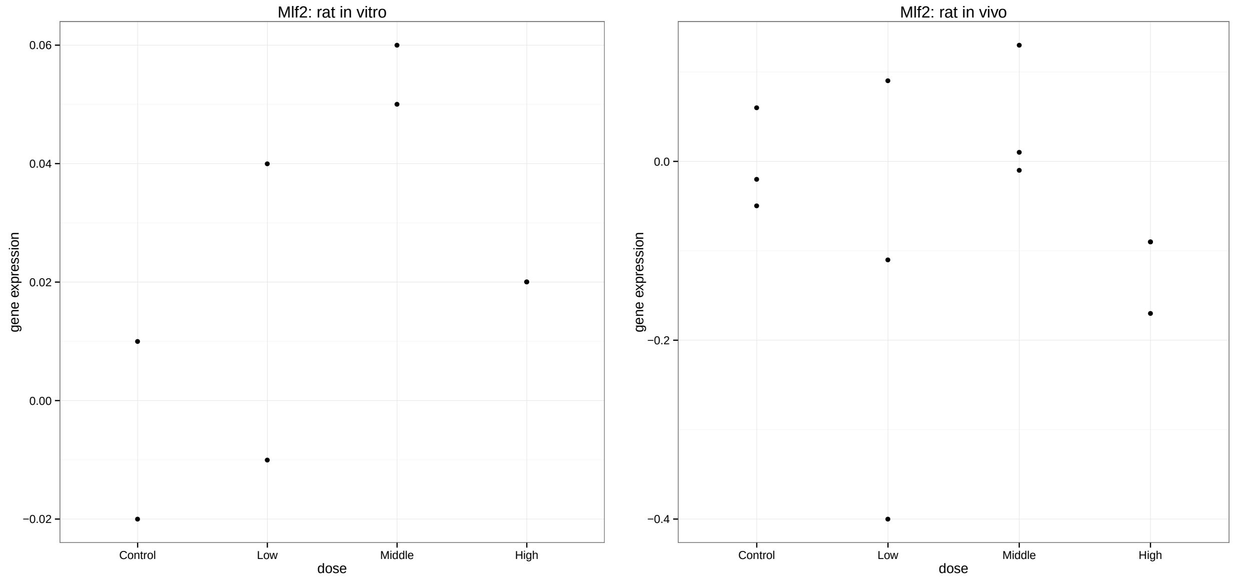

Figure 16: Compound diclofenac and gene *Mlf2*. Left panel: *in vitro*. Right panel: *in vivo*.

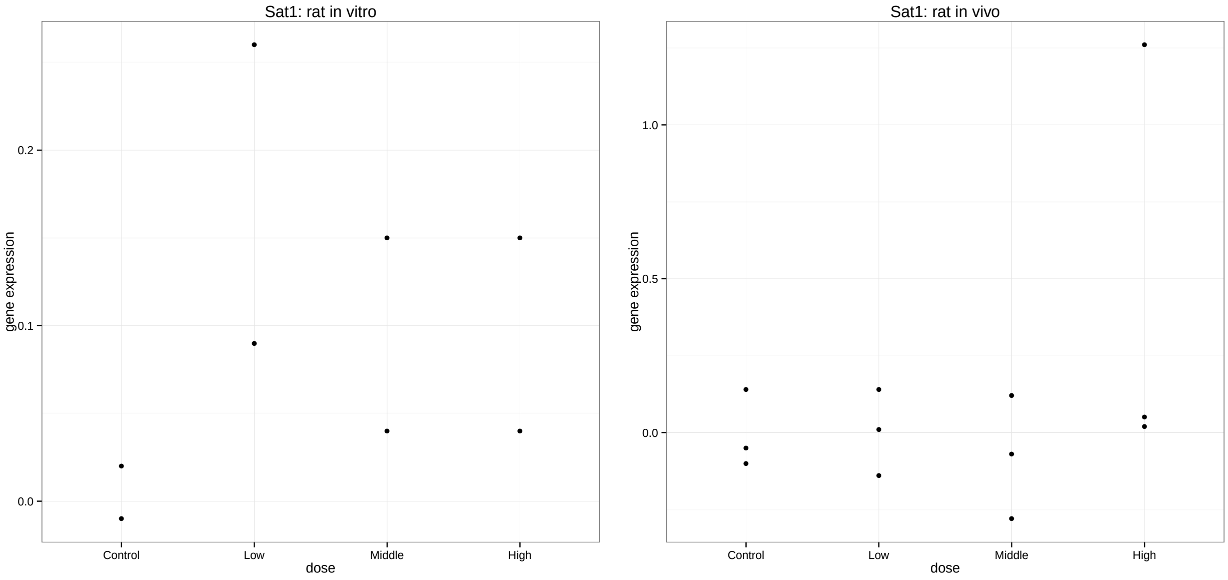

Figure 17: Compound diclofenac and gene *Sat1*. Left panel: *in vitro*. Right panel: *in vivo*.

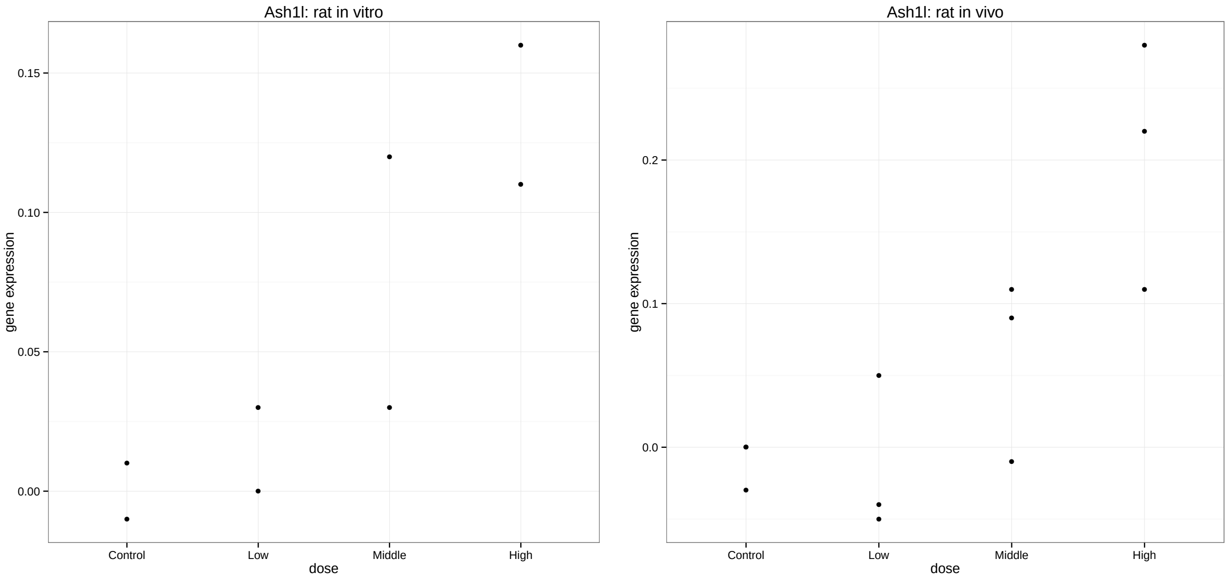

Figure 18: Compound diclofenac and gene *Ash1L*. Left panel: *in vitro*. Right panel: *in vivo*.

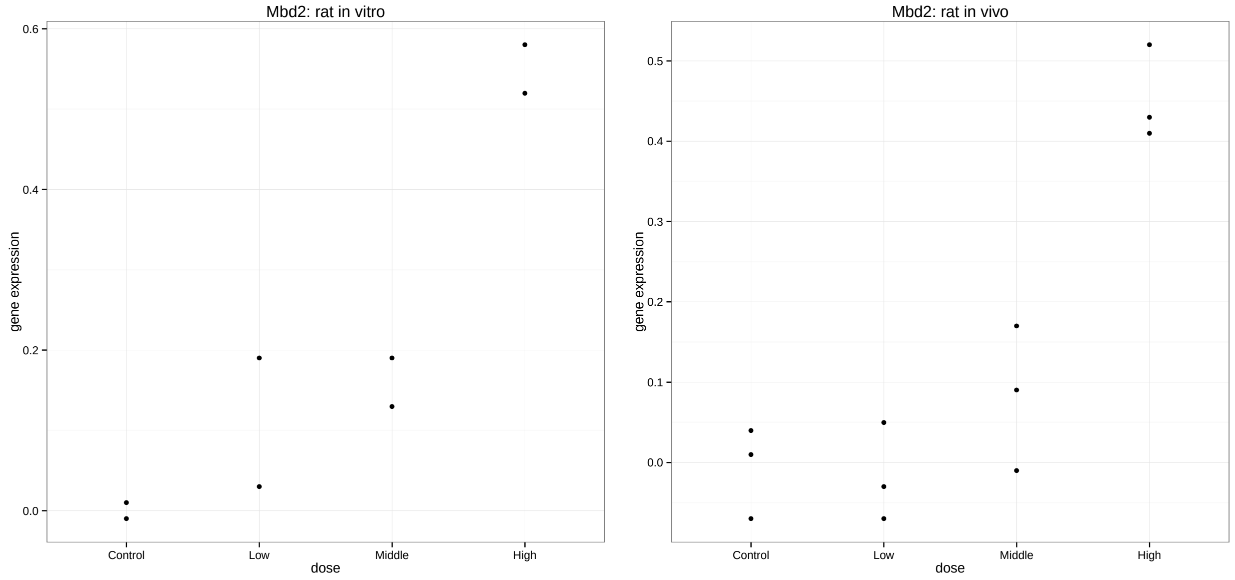

Figure 19: Compound diclofenac and gene *Mbd2*. Left panel: *in vitro*. Right panel: *in vivo*.

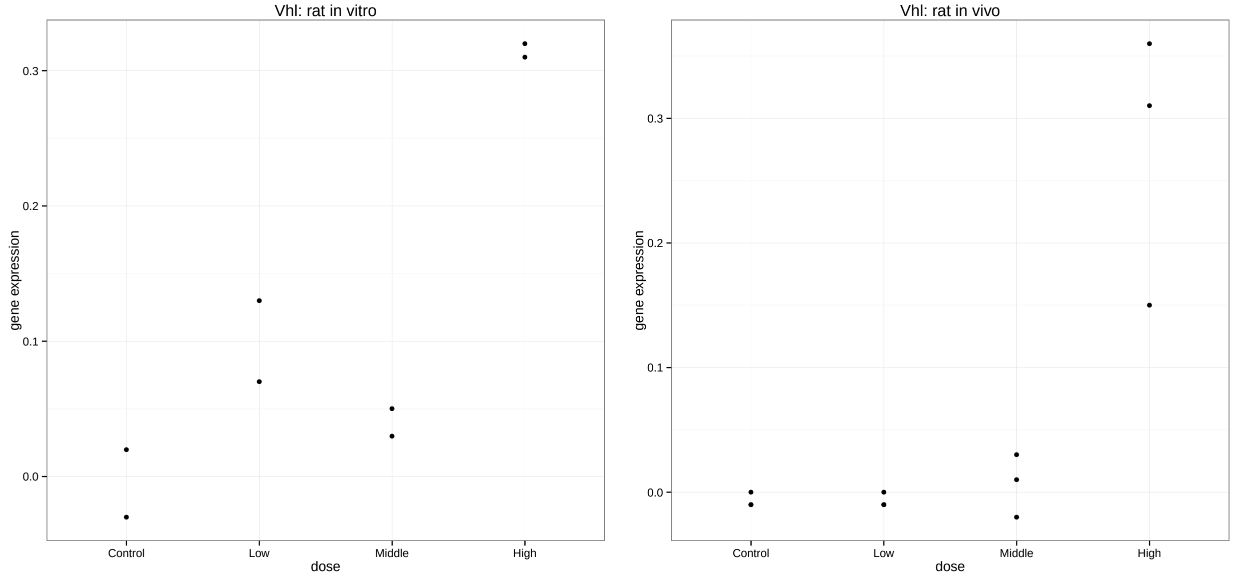

Figure 20: Compound diclofenac and gene *Vhl*. Left panel: *in vitro*. Right panel: *in vivo*.

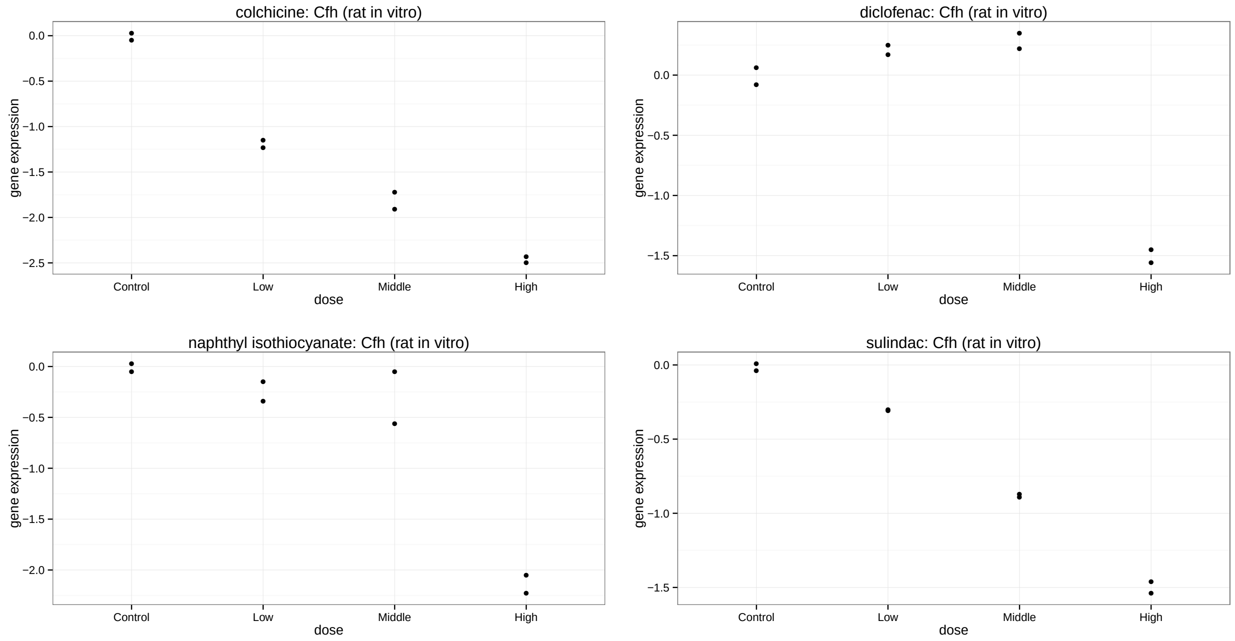

Figure 21: First bicluster when starting from *in vitro* data set. All four compounds that creates bicluster and gene *Cfh*.

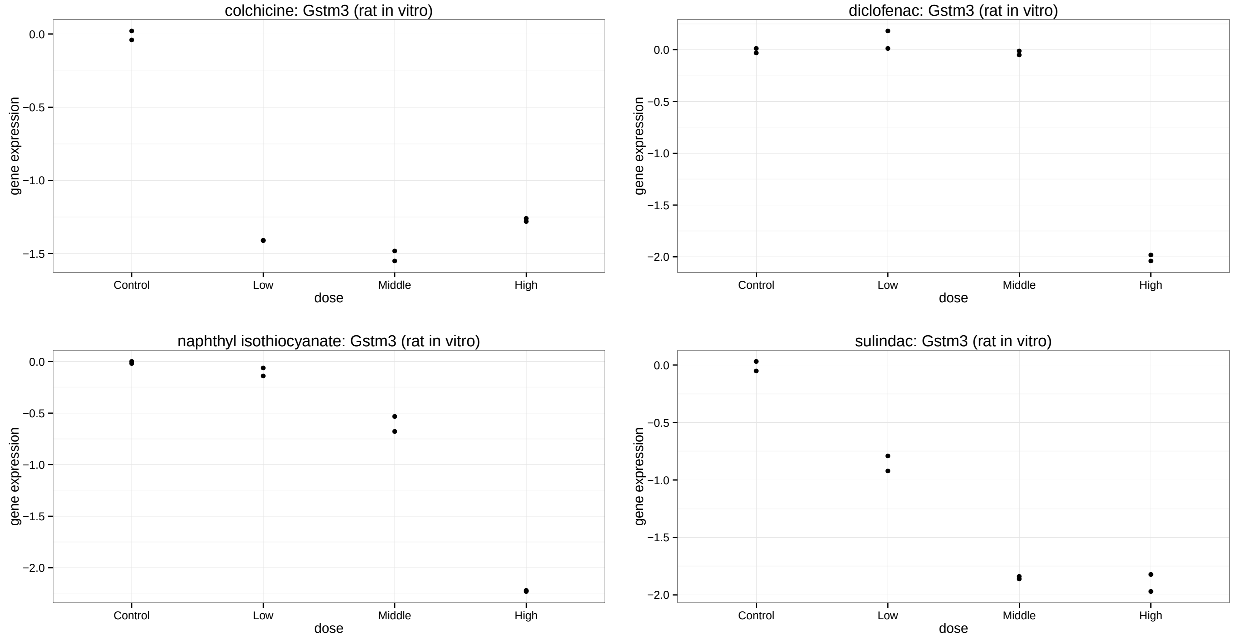

Figure 22: First bicluster when starting from *in vitro* data set. All four compounds that creates bicluster and gene *Gstm3*.

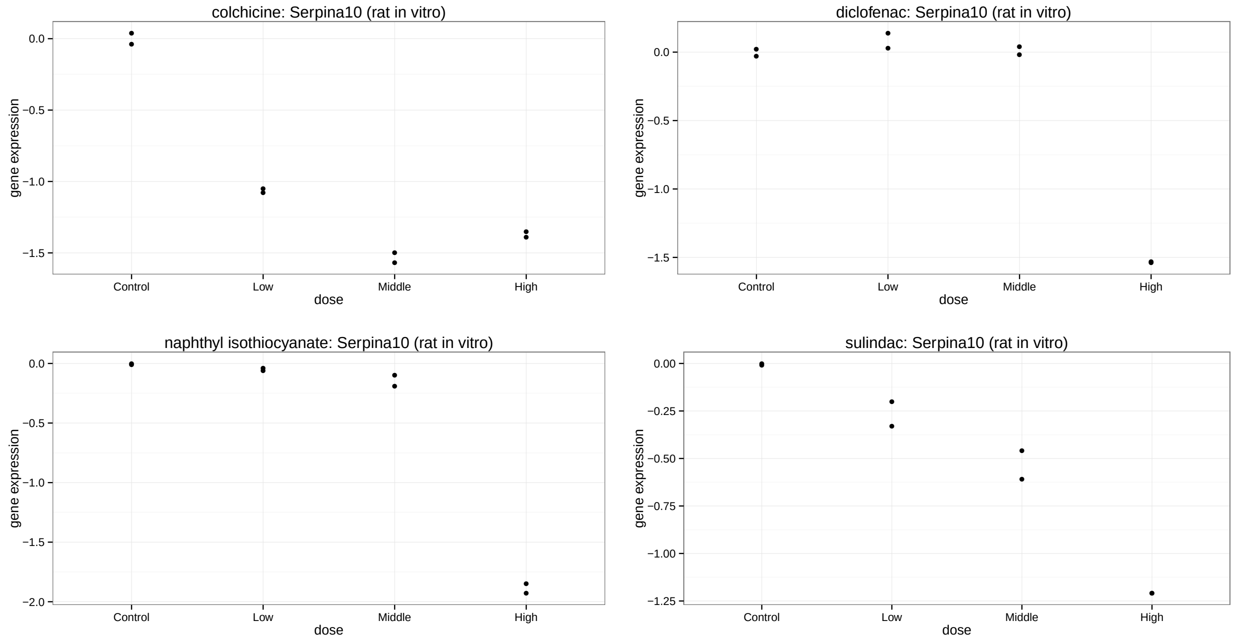

Figure 23: First bicluster when starting from *in vitro* data set. All four compounds that creates bicluster and gene *Serpina10*.

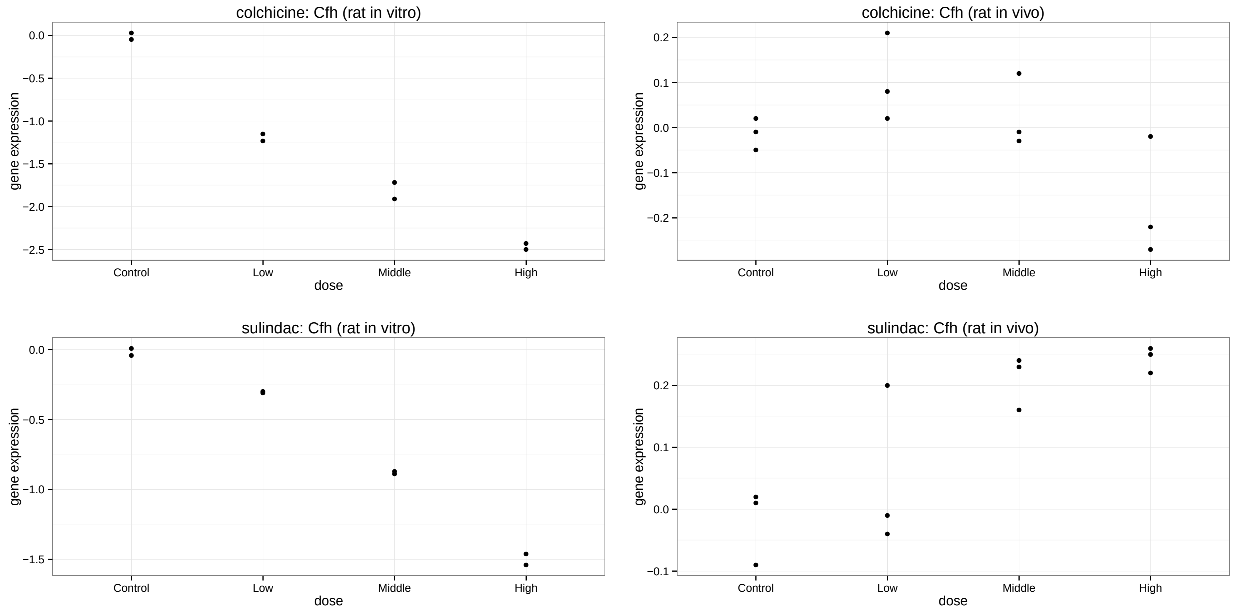

Figure 24: First bicluster when starting from *in vitro* data set. Compounds colchicine (top panels) and sulindac (bottom panels) and gene *Cfh*. Left panels: *in vitro*. Right panels: *in vivo*.

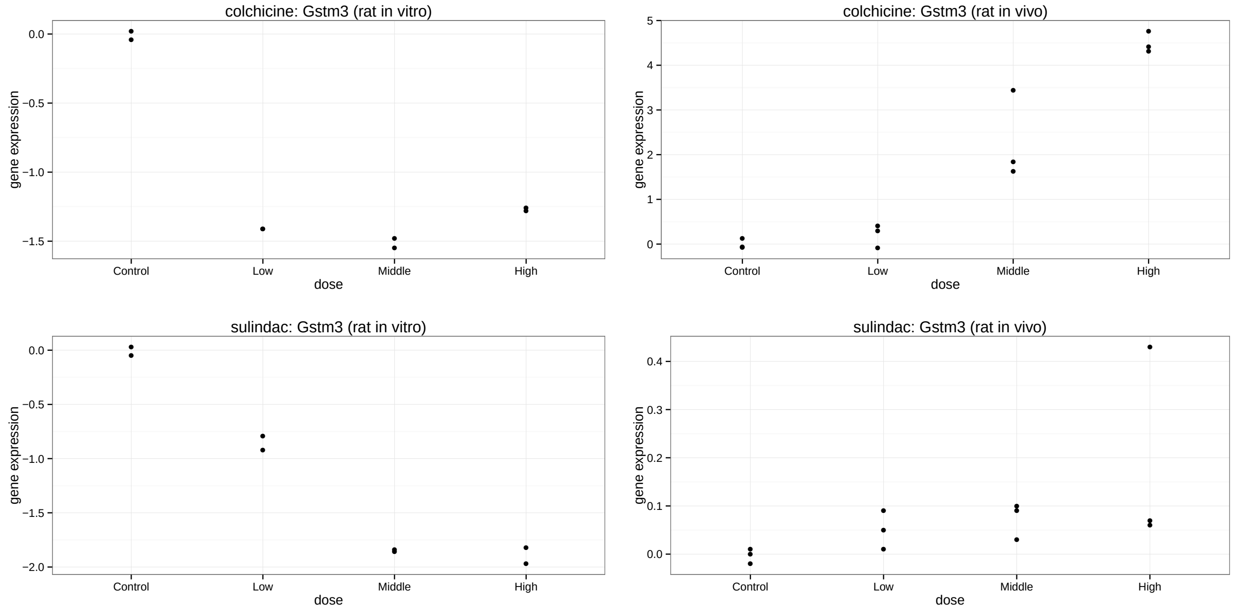

Figure 25: First bicluster when starting from *in vitro* data set. Compounds colchicine (top panels) and sulindac (bottom panels) and gene *Gstm3*. Left panels: *in vitro*. Right panels: *in vivo*.

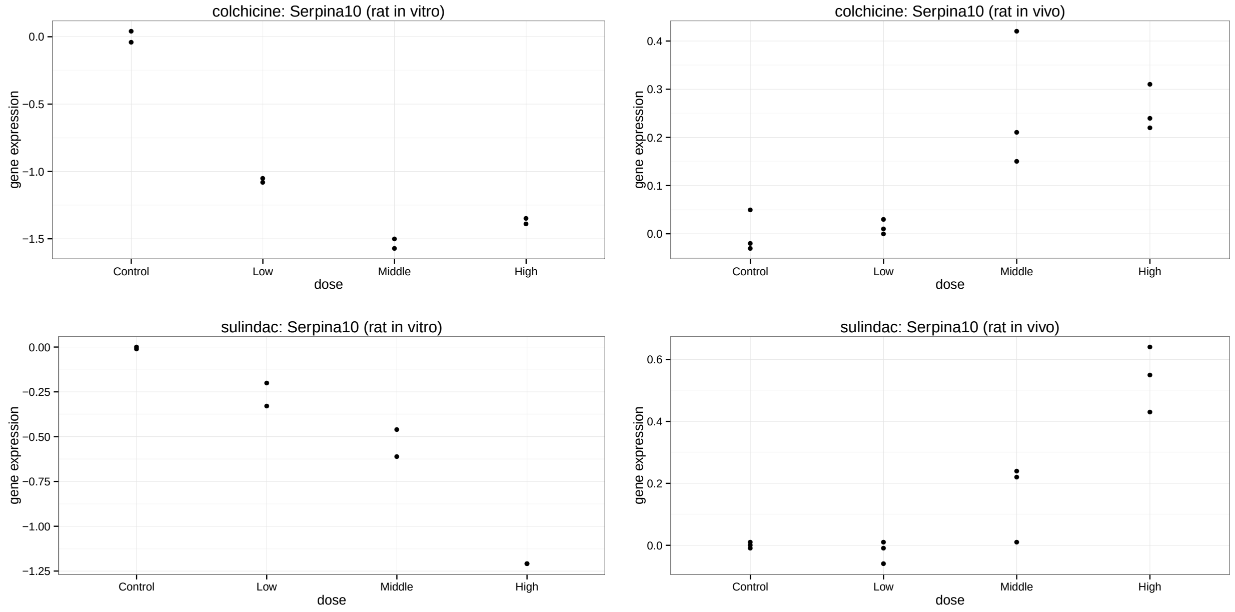

Figure 26: First bicluster when starting from *in vitro* data set. Compounds colchicine (top panels) and sulindac (bottom panels) and gene *Serpina10*. Left panels: *in vitro*. Right panels: *in vivo*.
